# Chondrocytes Embedded in Agarose Generate Distinct Metabolic Heat Profiles Based on Media Carbon Sources

**DOI:** 10.1007/s10439-025-03755-6

**Published:** 2025-06-01

**Authors:** Erik Myers, Priyanka Brahmachary, Sarah Mensah, Campbell Putnam, Ross P. Carlson, Mark Greenwood, Ronald K. June

**Affiliations:** 1 Department of Mechanical & Industrial Engineering, Montana State University, PO Box 173800, Bozeman, MT 59717-3800 USA; 2 Department of Mathematical Sciences, Montana State University, Bozeman, MT USA; 3 Department of Chemical and Biochemical Engineering, Montana State University, Bozeman, MT USA; 4 Department of Microbiology & Cell Biology, Montana State University, Bozeman, MT USA

**Keywords:** Chondrocyte, Metabolism, Osteoarthritis, Heat, Microcalorimeter

## Abstract

Human chondrocytes are responsible for cartilage repair and homeostasis through metabolic production of precursors to collagen and other matrix components. This metabolism is sensitive both to the availability of media energy sources as well as the local temperature. Central carbon metabolites such as glucose and glutamine are essential not only for producing energetic compounds such as ATP and NADH, but also for assembling collagen and aggrecan from non-essential amino acid precursors. The rate at which this metabolism takes place directly relates to temperature: a moderate increase in temperature results in faster enzyme kinetics and faster metabolic processes. Furthermore, these biological processes are exothermic and will generate heat as a byproduct, further heating the local environment of the cell. Prior studies suggest that mechanical stimuli affect levels of central metabolites in three-dimensionally cultured articular chondrocytes. But these prior studies have not determined if articular chondrocytes produce measurable heat. Thus, *the goal of this study* is to determine if three-dimensionally encapsulated chondrocytes are capable of heat production which will improve our knowledge of chondrocyte central metabolism and further validate in vitro methods. Here we show the results of microcalorimetric measurements of heat generated by chondrocytes suspended in agarose hydrogels over a 2-day period in PBS, glucose, and glutamine media. The results show that a significant amount of heat is generated by cells (Cells Only: 3.033 ± 0.574 µJ/cell, Glucose: 2.791 ± 0.819 µJ/cell, Glutamine: 1.900 ± 0.650 µJ/cell) versus the absence of cells (No Cells: 0.374 ± 0.251 µJ/cell). This suggests that cells which have access to carbon sources in the media or as intracellular reserves will generate a significant amount of heat as they process these metabolites, produce cellular energy, and synthesize collagen precursors. The length of the microcalorimeter experiment (48 h) also suggests that the metabolism of articular chondrocytes is slower than many other cells, such as human melanoma cells, which can produce similar quantities of heat in less than an hour. These data broadly suggest that chondrocyte metabolism is sensitive to the available nutrients and has the potential to alter cartilage temperature through metabolic activity.

**Supplementary Information:**

The online version contains supplementary material available at 10.1007/s10439-025-03755-6.

## Introduction

### Osteoarthritis is a Whole Joint Disease with Hallmark Cartilage Deterioration

Osteoarthritis (OA) is the most prevalent degenerative joint disease which results in losses of 1.8–3.5 quality-adjusted life years for patients [1]. OA pathophysiology affects tissues and cell types across the whole joint, most notably resulting in cartilage degradation including metabolic changes that affect the cell’s ability to maintain articular tissue homeostasis [2]. Chondrocytes are the only cell type in articular cartilage, responsible for matrix synthesis and production of new collagen and aggrecan precursors. Therefore, any impact to the health of these cells will result in cartilage damage over time. As chondrocyte matrix production is inhibited, matrix components are degraded and deteriorated at a rate greater than replacement matrix is synthesized [3].

### Osteoarthritis Involves Chondrocyte Metabolic Dysfunction

Articular cartilage health is directly linked to chondrocyte metabolic health. Dysregulation in metabolism limits chondrocyte metabolic function in OA [2]. Data show that chondrocytes are metabolically active in the use of both glycolysis and the tricarboxylic acid (TCA) cycle for energy and nonessential amino acid production [4, 5], suggesting that dysregulation or interruption of central glucose metabolism will also interrupt the necessary reparative functions of chondrocytes [6]. Recent data show impairment in the TCA cycle and OxPhos for OA chondrocytes contributing to the progression of OA by disrupting cellular energy production [7]. Additionally, several prior studies find that chondrocytes alter their metabolic activity in response to mechanical loading [8–12].

### Interconnection of Cartilage Heat and Chondrocyte Metabolic Activity

Central glucose metabolism produces heat as a byproduct of multiple exothermic chemical reactions [13]. This heat can affect surrounding cartilage matrix temperatures [14]. Temperatures above 41 °C cause chondrocyte death and dysfunction [15]. Furthermore, healthy chondrocyte metabolism is temperature-dependent, showing greatest production of non-essential amino acids and matrix precursors when temperatures are above normal joint values of 32 °C in the range of ~ 37–39 °C [16]. This suggests a connection between ideal temperature, chondrocyte metabolic activity, and heat generation. Previous experimental results find that mechanical stimuli induce changes in abundance of chondrocyte central metabolites [8–10, 12]. Computational models suggest that these changes support matrix production [11]. Yet, early studies suggest that chondrocyte metabolism in healthy cartilage is relatively inactive [17, 18]. Therefore, further experimental validation is needed to define the role of central metabolism in chondrocyte mechanotransduction. Hence, *the goal of this study* is to determine if three-dimensionally encapsulated chondrocytes are capable of measurable heat production and are thus potentially capable of modulating their local temperature. We hypothesized that in the presence of carbon sources chondrocytes would produce greater heat than without carbon sources due to metabolism of additional carbon.

## Methodology

### Experimental Components

**Fig. 1:**
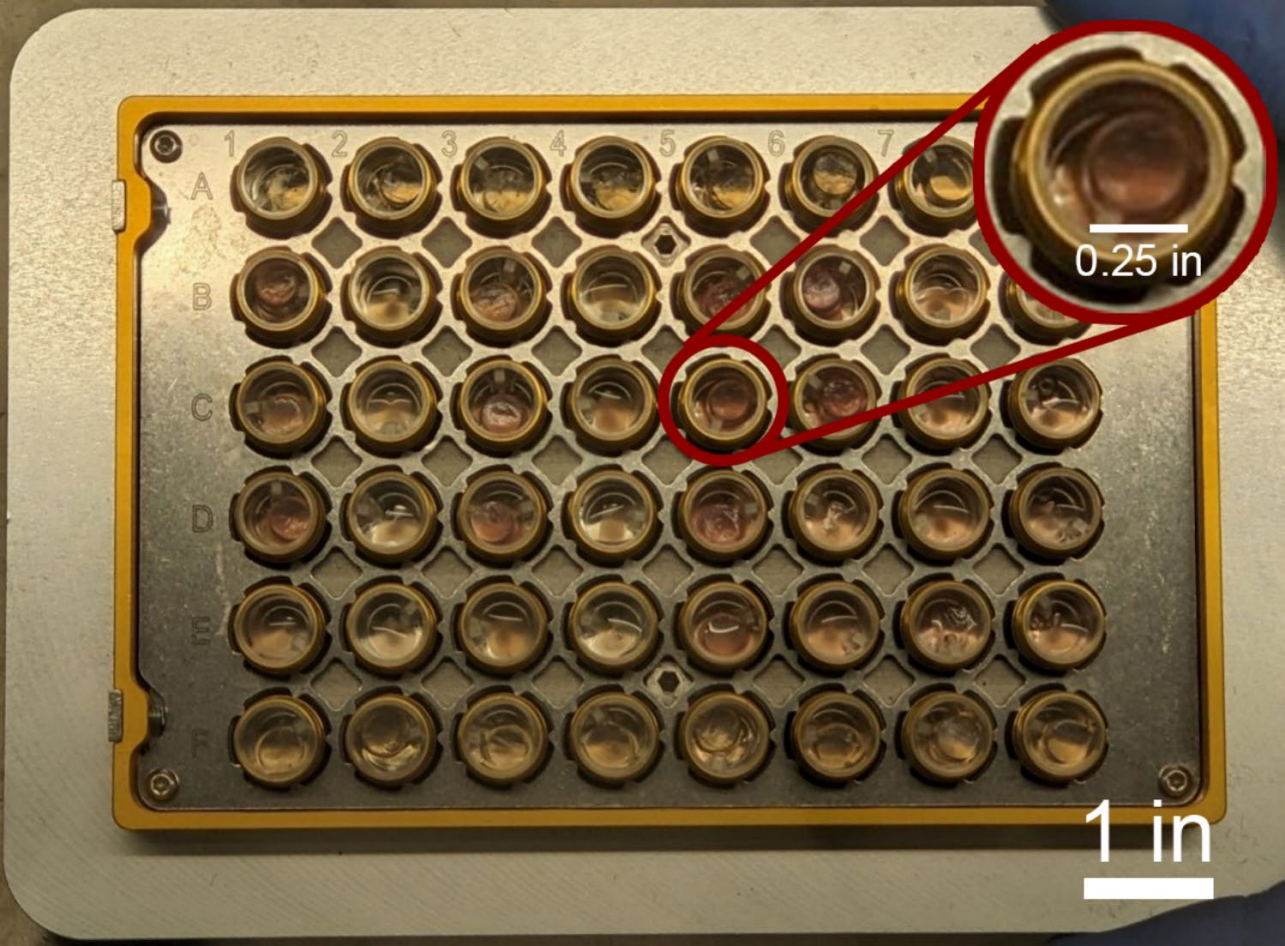
CalScreener sample cartridge with 48 wells and titanium capsules. Enlarged section highlights a single capsule with hydrogel cylinder inside, surrounded in media with scale bar indicating diameter of hydrogel

This study was performed using a microcalorimeter (CalScreener, SymCel, Stockholm, Sweden) with sufficient sensitivity to detect the small heat values (~ nW to µW) expected from articular chondrocytes [14]. The CalScreener is a self-contained, temperature controlled microcalorimeter that uses a cartridge holding up to 48 samples in individual titanium capsules, each with internal volume 656 µL. Capsules are hermetically sealed to isolate the specimen. The device requires 16 reference samples loaded on the top and bottom rows of the cartridge for calibration, resulting in 32 experimental samples per run. The sample cartridge is loaded horizontally into the CalScreener and allowed to reach equilibrium with the machine’s interior temperature before being aligned with individual thermocouples for data collection (Fig. 1).

After loading each capsule with a sample, the remaining volume is filled with media and air before sealing with a titanium lid. The CalScreener reads each column of samples and compares them to the references in that column, i.e., all samples in the first column (Samples B1, C1, D1, and E1) are referenced against the two references in the first column (References A1 and F1). Due to this, all samples in the same column must be comparable against the same reference. Since samples are hermetically sealed, capsules must have all required cell-seeded gels, media, and air before experimentation begins, meaning CO_2_ and oxygen must be accounted for before sealing. Initial data analysis was performed using CalView software.

### Chondrocyte Culture, Agarose Hydrogel Casting, and Media Suspension

Chondrocytes were harvested and cultured from either healthy bovine or human osteoarthritic cartilage using established protocols [19]. All samples were obtained with local ethics approval (Institutional Biosafety Committee for bovine samples and Institutional Review Board for human). Bovine cells were used exclusively for pilot studies, while human cells were used exclusively for the primary experimentation following pilot studies. Bovine samples were stifle joints from 18- to 22-month-old steers provided by a local abattoir (Feddes Family Meats, Amsterdam, MT). Human chondrocytes were isolated from discarded joint replacement tissue of a single 69-year-old male donor, obtained under IRB approval (Clinical trial number: not applicable), and 1st passage cells were used for all experiments. For each experiment, a single bovine or human donor was used to generate multiple replicates.

To provide an appropriate three-dimensional environment for chondrocytes, a stiff matrix media comprised of 4.95% weight/volume (wt/vol) low-gelling temperature agarose (Sigma-Aldrich A9045) was selected owing to its similar stiffness to the cartilage pericellular matrix [20]. Cells were mixed with low melting temperature agarose to give a final concentration of 4.5% wt/vol and allowed to solidify for 10 min, following which the agarose gels containing chondrocytes were placed in the sample wells. This agarose formulation also includes thermal properties which differ negligibly from articular cartilage and is ideal for the creation of a three-dimensional environment for chondrocyte cell culture [21]. Hydrogels were cast after autoclaving agarose powder for sterilization before mixing with cells and casting into an anodized aluminum mold. Once the samples were placed in capsules and hermetically sealed, the cartridge was immediately loaded into the microcalorimeter.

For this study, four different groups were compared in the same experimental run (Table 1): Group 1: No nutrient media (PBS only) and no chondrocytes (No Cells, NC), Group 2: No nutrient media (PBS only) plus chondrocytes (Cells Only, CO), Group 3: Media with glucose as the only carbon source and chondrocytes (Glucose, Glu), Group 4: Media with glutamine as the only carbon source and chondrocytes (Glutamine, Gln). 150,000 articular chondrocytes were embedded within each hydrogel. Hydrogels are 3/8” in height and 3/16” in diameter, dictated by the calorimeter chamber sizes. Pilot studies showed this number of cells was sufficient to provide a stable baseline signal (Supplemental Figs. 1–3, Supplemental Tables 1–3). A total of *n* = 8 sample gels was produced per group, for a total of 32 gels.

**Table 1:** Group names, cell counts, and suspension media across all four experimental groups

Experimental group	Chondrocytes per gel	Suspension media
No Cells (NC)	0	Phosphate-Buffered Saline (PBS)
Cells Only (CO)	150,000	Phosphate-Buffered Saline (PBS)
Glucose (Glu)	150,000	1 part (DMEM, 10% FBS, 25 mM HEPES, 1% PenStrep, 4.5 g/L Glucose, 110 mg/L Sodium Pyruvate) to 3 parts PBS
Glutamine (Gln)	150,000	1 part (DMEM, 10% FBS, 25 mM HEPES, 1% PenStrep, 2 mM Glutamine, 110 mg/L Sodium Pyruvate) to 3 parts PBS

All hydrogels were cast with DMEM, regardless of group

Nutrient enriched media were used both in the casting of the hydrogels as well as providing additional nutrients to the chondrocytes in the form of suspension media within the capsules. For the agarose hydrogel media, custom DMEM was prepared containing either 4.5 g/L glucose, or 2 mM l-glutamine, to which 110 mg/L sodium pyruvate, 10% FBS, 25 mM HEPES, and 1% PenStrep was added. The same batch of FBS was used for all experiments. Each hydrogel had a volume of approximately 150 µL. This left 506 µL of volume within the capsules to be divided between supplemental enriched media and air. 186 µL of media was added to each capsule. This resulted in an electron donor-to-acceptor ratio of ~ 5.5 for glucose-enriched media and ~ 2.6 for glutamine-enriched media, based on the elevation-corrected O_2_ concentration in the laboratory (~ 6.84 mmol O_2_ L-1). 186 µL of glucose-enriched media yielded an approximately stoichiometric electron balance between donors (glucose and pyruvate) and acceptor O_2_ (Supplemental Table 5). As such all capsules received 186 µL of solution regardless of composition. Suspension media allocation per group is illustrated in Table 1. DMEM used in suspension media was devoid of glucose and glutamine prior to formation. Control and reference capsules received identical supplemental media to the samples in each respective column, along with a hydrogel of the same media containing no cells. Two reference samples per column were needed for a total of 16 samples. A layout of all samples in the sample cartridge is available in the supplement (Supplemental Table 4).

### Microcalorimeter Data Collection

All cartridge and capsule components were either autoclaved or disinfected with UV and 70% ethanol 24 h prior to experimentation. On the day of experimentation, agarose hydrogels were cast using a bespoke aluminum mold and allowed to solidify for ~ 10 min at room temperature in a tissue culture hood. After gelation, samples were immediately extracted from the mold and sealed within each titanium capsule along with the appropriate media. After sealing, the cartridge was immediately inserted into the CalScreener, and data collection was initiated. Samples were allowed to equilibrate at 37 °C for ~ 30 min before being moved onto the thermocouples. The experiment was allowed to run until all heat generation signals from all samples had equalized around a steady-state value as close to zero as possible, at which point the samples were removed and the experiment was terminated. This period was 48 h in the case of the primary experiment and never exceeded 60 h in the pilot experiments.

### Data Processing and Statistical Analysis

**Fig. 2:**
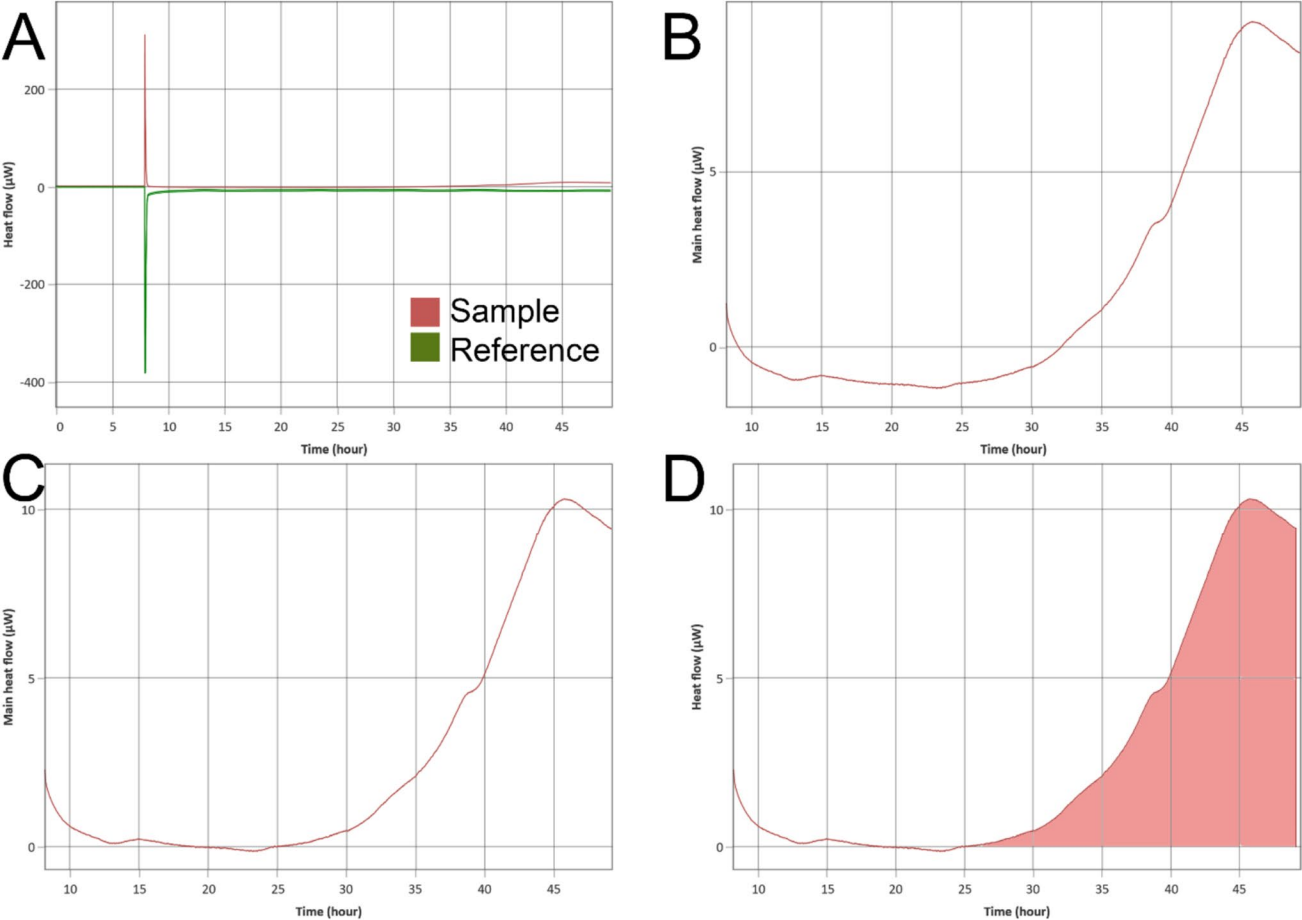
**A** A data signal from well D6 is automatically imported with its two reference signals from wells A6 and F6. Note the large spike in heat generation from the sample and reference wells at the beginning of the experiment, which results from the friction of loading the cassette into the CalScreener. **B** The reference signals are averaged and subtracted from the heat generation signal from well D6. The extraneous time periods at the beginning and end of the run are removed to form the “Main” signal. **C** Baseline correction then maintains heat generation values as positive during the “Main” signal. **D** “Main” signal can then be integrated to gather total heat generation value

Heat generation signals were sampled at 1 Hz for the duration of the experiment, which lasted approximately 48 h. Note that negligible cell division is expected during this time period for articular chondrocytes grown in three dimensions [22]. Signals from each capsule were then corrected by subtracting the average reference signal within the CalView software (Fig. 2). Each signal was then trimmed to exclude the loading procedure at the beginning of the experiment and extraneous steady-state data at the end of the experiment. These signals were then isolated, and each baseline adjusted for column-wise drift to maintain positive signal for the length of the experiment. The remaining signal was then analyzed for differences in the instantaneous heat generation values over the course of the experiment as well as differences in the total heat generated.

All statistical analyses were completed using the statistical software R [23] using total heat generation as the dependent variable. The rationale for using total heat generation is that known values of heat are released from metabolism of specific media carbon sources [24]. The variances were markedly different between the groups on total heat, so a generalized least squares (GLS) model that assumes different variances for each group was used and pairwise comparisons were performed using Tukey's Honest Significant Difference to determine which groups differed in average total heat using that GLS model [25–27].

## Results

### Instantaneous and Total Heat Generation Differences

**Fig. 3:**
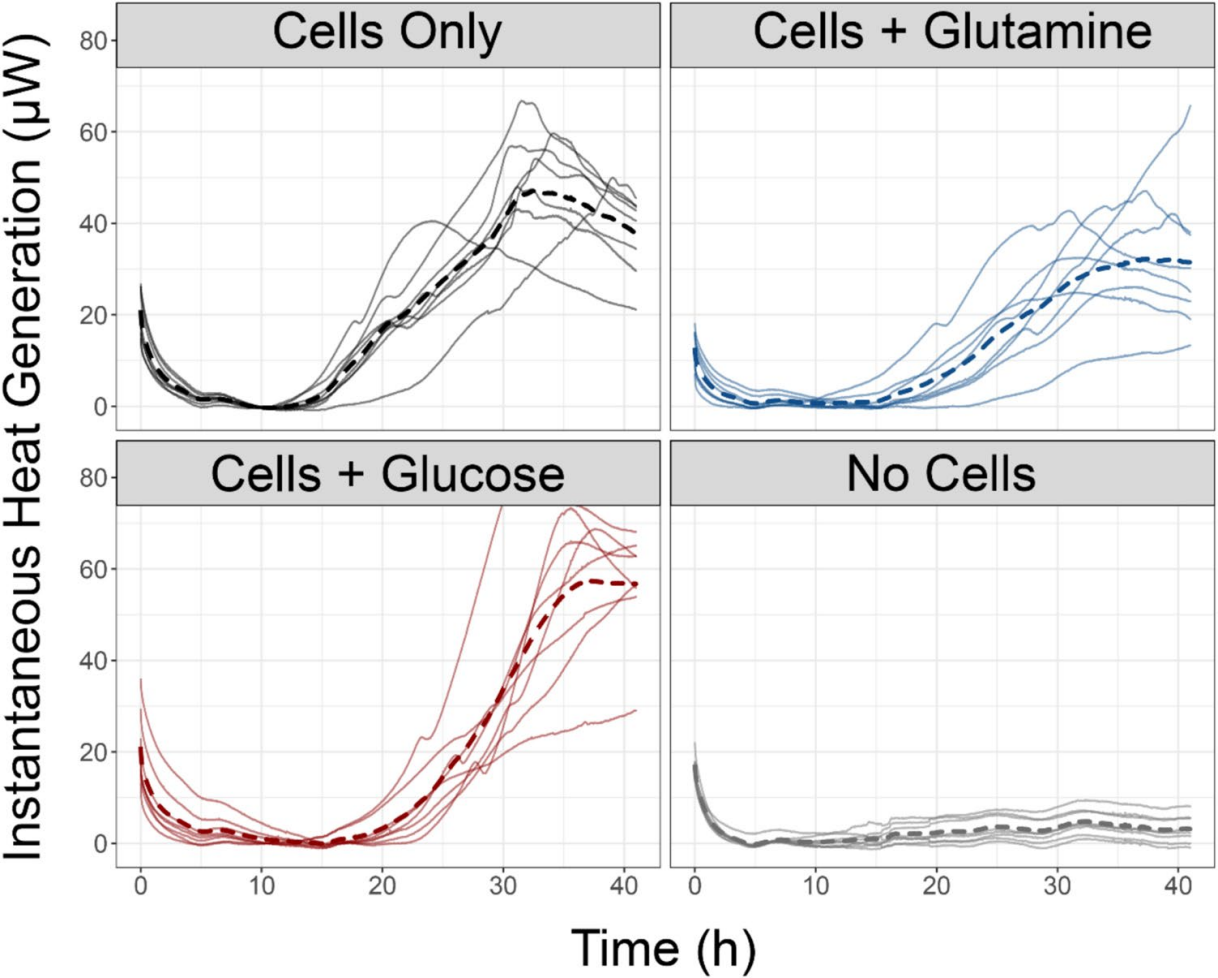
Instantaneous heat generation data demonstrates an increase in heat output in samples containing chondrocytes. Data for each group include each individual sample trace in a solid line, and the group mean in a dashed bold line

Following the 48 h experimental run, the instantaneous heat generation profiles were analyzed by group versus time (Fig. 3).

Instantaneous heat generation typically displayed an initial decrease after cartridge loading followed by relatively low values until samples with chondrocytes began generating heat after ~ 12 h, depending on media. The initial downward trend in heat in the 0–5-h span represents dissipation of the residual heat generated from friction during loading of the cartridge within the machine as seen in prior studies [28] and confirmed by personal communications with Symcel engineers. Values reaching near-zero in the ~ 10-h mark represent the baseline offset area, where each signal was adjusted to remain positive during the experimental run. The remaining period post-10 h represents heat generated by the samples within the capsules. Signals generally settled to the baseline after approximately 5–7 h and began generating heat signals above the baseline after 12–15 h. These values held or increased for the remainder of the experiment. The individual instantaneous heat generation values from Fig. 3 were integrated with respect to time to find the total heat energy output by each sample (Table 2). Total heat generation values were normalized by the number of cells.

**Table 2:** Integrated heat generation profiles show total heat generated over the course of 48 h

Cell ID	Group	Total heat (µJ/cell)	Cell ID	Group	Total heat (µJ/cell)
B1	No Cells	0.25	B5	Cells + Glucose	1.54
B2	No Cells	0.74	B6	Cells + Glucose	2.65
C1	No Cells	0.03	C5	Cells + Glucose	2.08
C2	No Cells	0.56	C6	Cells + Glucose	4.34
D1	No Cells	0.31	D5	Cells + Glucose	3.03
D2	No Cells	0.18	D6	Cells + Glucose	2.61
E1	No Cells	0.66	E5	Cells + Glucose	3.11
E2	No Cells	0.25	E6	Cells + Glucose	2.98
B3	Cells Only	3.26	B7	Cells + Glutamine	1.42
B4	Cells Only	3.45	B8	Cells + Glutamine	1.82
C3	Cells Only	2.83	C7	Cells + Glutamine	0.68
C4	Cells Only	3.97	C8	Cells + Glutamine	1.77
D3	Cells Only	3.00	D7	Cells + Glutamine	2.37
D4	Cells Only	3.00	D8	Cells + Glutamine	2.07
E3	Cells Only	2.76	E7	Cells + Glutamine	2.23
E4	Cells Only	1.99	E8	Cells + Glutamine	2.83

**Fig. 4:**
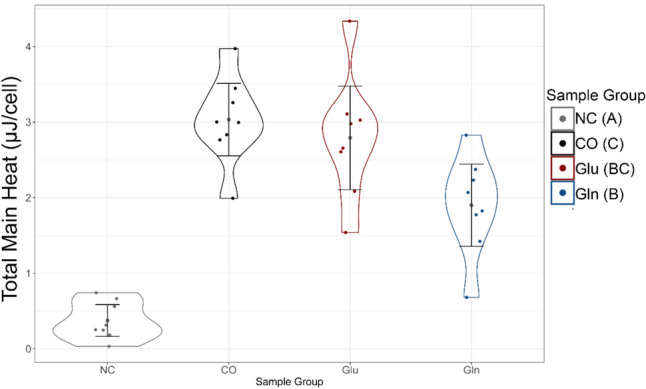
Enhanced stripchart of total heat generation values reveals distributions of individual heat generation values. Bars within strip areas represent the 95% confidence interval of the group. Sample groups are labeled with Compact Letter Display group in parentheses which indicates statistical differences between groups without shared letters.

The total heat generation data (integrated from Fig. 3) show clear differences between cell-encapsulated groups and no-cell controls (Fig. 4). More detailed representations of this data can be found in the supplement using emmeans [27, 29] (Supplemental Material 1).

### Statistical Analysis of Total Heat Generation

To combat the observed heteroscedasticity across groups (Fig. 4), a statistical model was fit using Generalized Least Squares (GLS) that allowed different variances for each group for the total heat response. Group differences were analyzed using an ANOVA *F*-test and Tukey’s Honest Significance Difference pairwise comparisons between groups [25, 26].

There was strong evidence of a difference in mean total heat [*F*(3, 28) = 67.8, *p* < 0.0001] from this model. The no-cell control had the smallest estimated mean total heat and was different from all other groups at the 5% family-wise significance level. The CO group (containing cells and no supplemental glucose or glutamine) had the highest estimated mean and was also detectably different from the group containing glutamine-enriched media. The groups with either glucose or glutamine media were not detectably different from each other, but the group containing glucose media did have a higher estimated mean total heat generation. Models that accounted for the row in the cartridge did not show systematic variation in the total heat by row, so are not discussed, although enhanced stripcharts of the responses based on cartridge row are available in the supplement (Supplemental Material 1). Because of the required configuration of the reference samples, it is not possible to disentangle the variation in the columns of the array from the treatments applied. It is theoretically possible that the variation in the groups could be partially due to the column-wise organization of the cartridge. However, pilot studies suggest that column-wise variation in heat generation values is negligible. Further details on the statistical modeling and results of pilots can be found in the supplemental material.

## Discussion

### Heat Generation of Chondrocytes is Slow and Stable

The generation of heat from chondrocytes appears to be dependent on several factors (Figs. 3 and 4). Firstly, significant heat generation values from these cells do not appear immediately when placed within the capsules. As seen in pilot studies and the primary 48-h study, there is an interim period during which chondrocytes do not generate heat above baseline levels. In most cases, there is a delay of between 12 and 24 h during which no heat is generated, regardless of enriched media presence. This is most likely due to the period of time in which recently embedded cells are acclimating to their surroundings [30]. After the cells have had an appropriate acclimation period, they begin metabolizing carbon from either intracellular reserves or from the media which may generate precursors to non-essential amino acids for production of matrix proteins like collagen. Due to this delay in metabolic activation, all samples showed approximately the same pause prior to initiation of heat generation, which was independent of media composition. Information regarding other pilot study heat generation values is available in the supplement (Supplemental Figs. 1–3). Contrary to our hypothesis, there were no differences in heat production between chondrocytes in the presence or absence of media carbon sources. We attribute this to the presence of intracellular carbon stores that allow chondrocytes to produce heat without external carbon sources. The storage and usage of intracellular carbon stores is likely related to the nutrient poor, avascularized environment of articular cartilage.

Chondrocyte heat generation was generally consistent within all media samples, with very little evidence of large fluctuations in heat generation values apparent in instantaneous readings. Chondrocytes also produced peak heat values within 48 h of experiment start; a trend which likely relates to both the embedding media and three-dimensional diffusion required for O_2_ to move into the hydrogel. This is in stark contrast to metabolism of other systems like human melanoma cells, which can generate similar heat values in less than an hour [31]. The magnitude of chondrocyte heat production was also within expected values of 4–50 pW/cell, which was similar to reported heat generation values of murine neuroblastoma cells, around 35 pW/cell [14]. This suggests that chondrocytes may be similarly efficient in their utilization of available media compared to the prior data from mammalian neuroblastoma cells. It remains unclear how chondrocyte heat production might vary during amino acid precursor production or collagen synthesis after the cells have reached environmental equilibrium.

### Chondrocyte Heat Generation Data Clearly Show Metabolic Activity

Because prior experiments found that chondrocyte central metabolite levels changed in response to mechanical stimuli [8–10, 12], the goal of this study was to determine if three-dimensionally encapsulated chondrocytes are capable of measurable heat production. Clearly samples including chondrocytes produced significantly more heat than cell-free agarose samples. These data suggest that chondrocytes can produce measurable heat, on the order of millijoules for 150,000 cells. The articular cartilage in human joints contains millions of chondrocytes, and the model of cartilage as an effective thermal battery with a relatively large heat capacitance and low thermal conductivity suggest that some heat generated by chondrocytes during metabolic activity may reside temporarily in the joint in the form of temperature increase [8, 10]. This temperature increase might then further alter the chondrocyte metabolism, turning the joint into a well-functioning bioreactor which is sensitive to surrounding media and O_2_ tension [3].

The specific carbon source may also determine heat generation values (Fig. 2), although further research here is needed. There is an initial spike in the heat generated by the Cells Only group. The peak heat generation of ~ 48 pW/cell is most likely the result of cells processing stored carbon sources, before exhausting their fuel source at around 32 h. Catabolism of stored energy sources was comparable with externally added energy sources for 48 h. Because chondrocytes survive in an avascular environment, their ability to sustain heat production for this amount of time suggests they have a substantial reserve to buffer perturbations in carbon source availability.

In contrast to this, the glucose and glutamine groups both generated more heat late-term in the experiment, reaching peak values around the 38-h mark and holding these values until the conclusion of the experiment. While there was no statistically significant difference in the total heat generated between the glucose and glutamine groups (Fig. 4, ~ 3 vs 2 mJ, respectively), the instantaneous heat generation values shown in Fig. 3 were different during peak heat generation times. This is most likely because metabolism of glucose and glutamine have different end points. For example, complete oxidation of the carbons in glucose to carbon dioxide results in more heat release than metabolism of glutamine to form amino acid precursors [13]. This may explain why glucose and glutamine groups showed stable heat generation totals which were not statistically significant from one another, but the instantaneous peak remained distinct.

### Chondrocyte Health and Concentration has the Potential to Affect Joint Temperature

Cartilage may be considered as a thermal battery, with a high specific heat capacity and low thermal conductivity when compared to other bodily tissues (3.20 J/gK and 0.21 W/mK, respectively) [32]. This means that heat generated in the tissue can be stored temporarily as a temperature increase, raising the overall joint temperature. In some cases of strenuous exercise, joint temperature can increase by as much as 6 °C [33]. This heat generation may be due in part to the metabolic activity of the chondrocytes.

As such, the metabolically generated heat and subsequent joint temperature change may be directly related to both the number of chondrocytes present in the joint as well as the available energy sources and metabolic activity of the cells in processing carbon sources. Danalache et al. found that cell density decreased by 63% in the superficial zone between healthy and osteoarthritic human cartilage, with an average 50% decrease in cell count in single coherent strings of healthy chondrocytes throughout the cartilage depth [34]. Similarly, Allen et al. recorded a decrease of 50–70% in viable cell density of articular chondrocytes between young and aging rabbits. For the cell density changes reported in Danalache et al. of 4363 cells/mm^2^ in healthy human superficial cartilage down to 1622 cells/mm^2^ in OA human cartilage, this could result in a decrease of generated heat per unit area from 12.18 mJ/mm^2^ down to 4.53 mJ/mm^2^. When coupled with the fact that OA cartilage shows decreased thickness when compared to healthy cartilage (3.9 mm decreasing to 3.68 mm in the central medial femorotibial compartment [35]), this results in a compounding reduction in heat generated per unit depth in the sagittal plane from 50.4 mJ/mm down to 16.7 mJ/mm; a 67% decrease in overall heat generated per unit depth (Supplemental Equation 1). Although this estimate is limited by the depth dependence of cartilage cell density, the major projected reduction in heat generation capacity might directly affect the joint environment through a feed-forward mechanism: decreased temperatures may result in further decreases in chondrocyte metabolic activity which then further decrease joint temperature. Future studies are needed to further examine this potential mechanism.

The data resulting from these microcalorimeter experiments yields novel insight regarding the heat generated by healthy chondrocytes when exposed to enriched media which is used in their central carbon metabolism. These data clearly demonstrate that chondrocytes suspended in a 3-D structure mimicking their in vivo pericellular matrix stiffness are metabolically active and capable of generating heat analogous to in vivo chondrocytes (Figs. 3 and 4, Table 2). The heat generated in these cells is similar in magnitude to murine neuroblastoma cells [14]. However, there is limited information available for comparison since there are few studies on heat production measurements in mammalian cells.

There are limitations to this study. The equilibration time for chondrocytes after embedding might have influenced these results, as chondrocytes may need additional time to adjust to encapsulation in agarose. We did not measure viability at the conclusion of the experiments. Future experiments might compare heat generation across several additional effects including donor, sex, OA grade of cartilage donor, oxygen tension, and potential physical or environmental stimulants of chondrocyte metabolism and production of matrix components. 

In summary, heat production of chondrocytes encapsulated in 3-D agarose hydrogels follows a reliable pattern based on metabolic activity of the chondrocytes. An initial dormancy phase likely based on acclimation to the new environment was followed by remarkably repeatable heat generation trends as cells utilized available oxygen and nutrients within the medium. These results demonstrate that chondrocytes are metabolically active when embedded in physiologically stiff agarose matrix as demonstrated by the generation of measurable heat. This thermal output stimulated by different carbon sources can serve as a valuable indicator of cellular metabolic activity and lead to a better understanding of basic chondrocyte biology. 

## Supplementary Information

Below is the link to the electronic supplementary material.

Supplementary file1 (PDF 5063 KB)
