# Supplementary material for: Chondrocytes Embedded in Agarose Generate Distinct Metabolic Heat Profiles Based on Media Carbon Sources
Source: Ann Biomed Eng. 2025 Jun 1;53(9):2071–9. doi: 10.1007/s10439-025-03755-6 (PMC12391224; doi:10.1007/s10439-025-03755-6)
Supplement: Supplementary file 1 — Supplementary file1 (PDF 5063 KB) [file 10439_2025_3755_MOESM1_ESM.pdf]

Supplemental Material

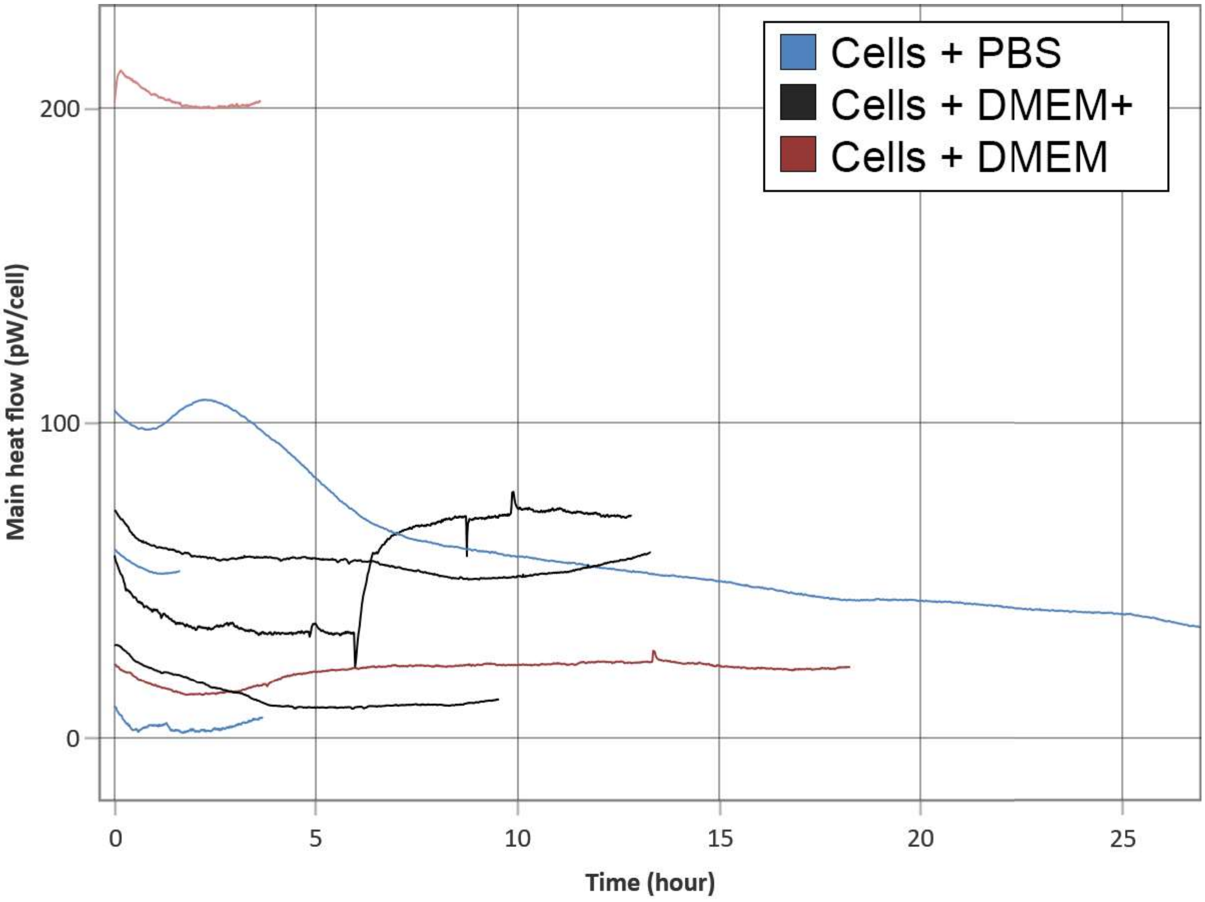

Supplemental Figure 1: Mean instantaneous heat generation values normalized per cell. This 1st microcalorimeter experiment used low cell concentrations of 15k, 30k, and 50k cells per agarose hydrogel. Signals generated here were filled with noise, and many individual samples suffered from bacterial contamination overruling heat generated by the limited number of chondrocytes.

Supplemental Table 1: Cartridge layout of initial pilot microcalorimeter experiment. The goal of this experiment was to determine if 50,000 chondrocytes could generate a consistent signal in monolayer culture. This experiment showed as many as 50,000 chondrocytes could not generate a consistent signal from the microcalorimeter. Instrument blank controls are in rows A and F and align with their respective columns.

	**1**	**2**	**3**	**4**	**5**	**6**	**7**	**8**
**A**	PBS$_C$1	PBS$_C$2	PBS$_C$3	PBS$_C$4	DMEM$_C$1	DMEM$_C$2	DMEM$_C$3	DMEM$_C$4
**B**	15k Cells + Plain DMEM 1	15k Cells + Plain DMEM 2	15k Cells + Plain DMEM 3	15k Cells + Plain DMEM 4	30k Cells + Plain DMEM 1	30k Cells + Plain DMEM 2	30k Cells + Plain DMEM 3	30k Cells + Plain DMEM 4
**C**	15k Cells + PBS 1	15k Cells + PBS 2	15k Cells + PBS 3	15k Cells + PBS 4	15k Cells + DMEM 1	15k Cells + DMEM 2	15k Cells + DMEM 3	15k Cells + DMEM 4
**D**	30k Cells + PBS 1	30k Cells + PBS 2	30k Cells + PBS 3	30k Cells + PBS 4	30k Cells + DMEM 1	30k Cells + DMEM 2	30k Cells + DMEM 3	30k Cells + DMEM 4
**E**	50k Cells + PBS 1	50k Cells + PBS 2	50k Cells + PBS 3	50k Cells + PBS 4	50K Cells + DMEM 1	50K Cells + DMEM 2	50K Cells + DMEM 3	50K Cells + DMEM 4
**F**	PBS$_C$5	PBS$_C$6	PBS$_C$7	PBS$_C$8	DMEM$_C$5	DMEM$_C$6	DMEM$_C$7	DMEM$_C$8

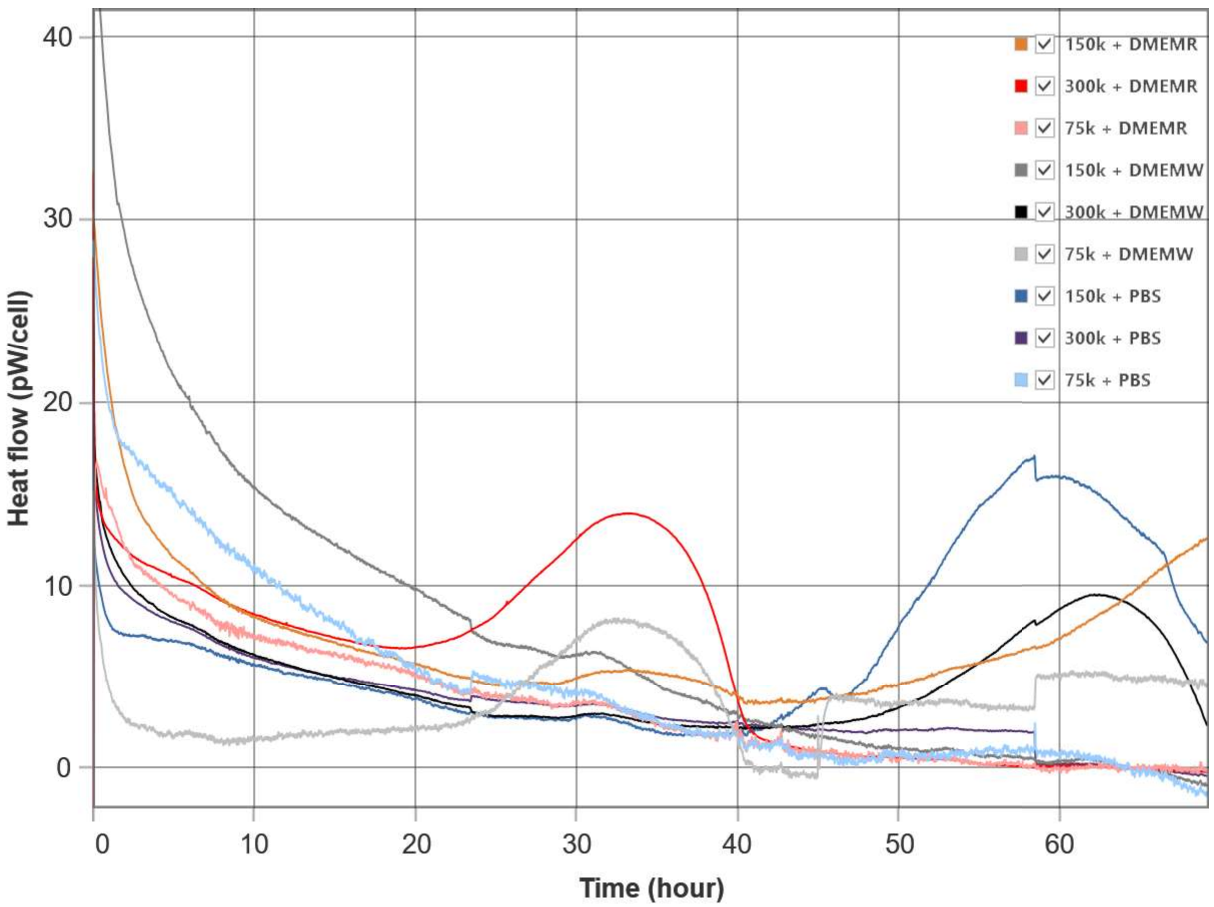

Supplemental Figure 2: Second pilot experiment using agarose hydrogels. Mean instantaneous heat generation values normalized per cell. This 2nd microcalorimeter experiment used 75k, 150k, and 300k chondrocytes per hydrogel, as well as Three different types of media: PBS, Readily-available DMEM with Phenol Red (DMEMR), and In-house prepared DMEM (DMEMW). Heat generation values shown here are well enough above the baseline of noise to suggest that a cell concentration of at least 150,000 cells per gel is great enough to gather meaningful results.

Supplemental Table 2: Cartridge layout of second pilot experiment. DMEMW (DMEM White) has 25mM HEPES,10% FBS, and 1% PenStrep added. DMEMR (DMEM Red) Has 24mM HEPES added. The agarose gels have 1mL cells in DMEM with PenStrep, no FBS. These cells were healthy bovine cells, unlike all other experiments which used OA human cell lines.

	**1**	**2**	**3**	**4**	**5**	**6**	**7**	**8**
**A**	PBS$_C$1	PBS$_C$2	DMEMW$_C$1	DMEMW$_C$2	DMEMR$_C$1	DMEMR$_C$2	DMEMR$_C$3	DMEMR$_C$4
**B**	300k + PBS<br>1	150k + PBS<br>2	300k + DMEMW<br>1	150k + DMEMW<br>2	300k + DMEMR<br>1	300k + DMEMR<br>5	150k + DMEMR<br>3	75k + DMEMR<br>2
**C**	300k + PBS<br>2	150k + PBS<br>3	300k + DMEMW<br>2	150k + DMEMW<br>3	300k + DMEMR<br>2	300k + DMEMR<br>6	150k + DMEMR<br>4	75k + DMEMR<br>3
**D**	300k + PBS<br>3	75k + PBS<br>1	300k +DMEMW<br>3	75k + DMEMW<br>1	300k + DMEMR<br>3	150k + DMEMR<br>1	150k + DMEMR<br>5	75k + DMEMR<br>4
**E**	150k + PBS<br>1	75k + PBS<br>2	150k + DMEMW<br>1	75k + DMEMW<br>2	300k + DMEMR<br>4	150k + DMEMR<br>2	75k + DMEMR<br>1	75k + DMEMR<br>5
**F**	PBS$_C$3	PBS$_C$4	DMEMW$_C$3	DMEMW$_C$4	DMEMR$_C$5	DMEMR$_C$6	DMEMR$_C$7	DMEMR$_C$8

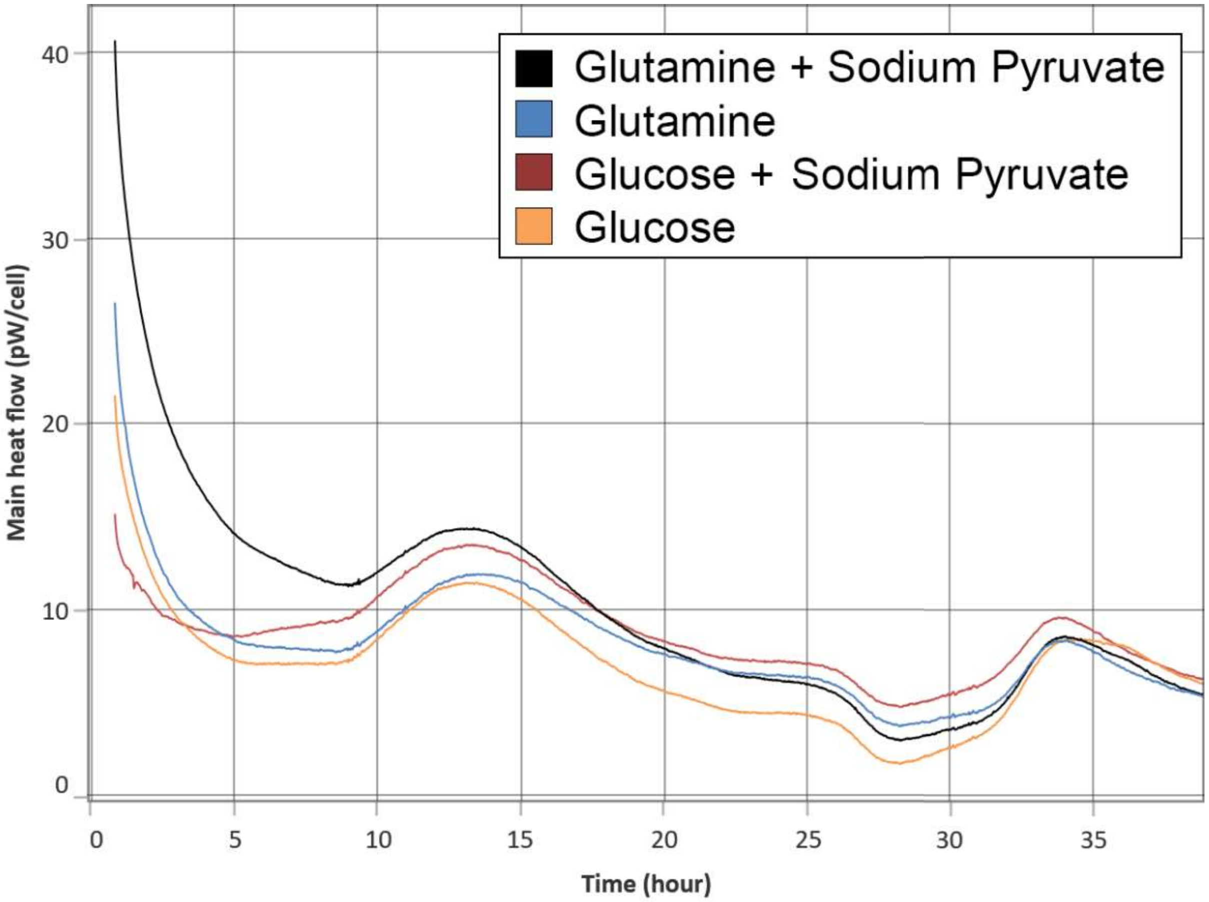

Supplemental Figure 3: Mean instantaneous heat generation values normalized per cell. This 3rd microcalorimeter experiment used only one cell density of 150,000 cells per hydrogel, given the results of the prior experiment. Available media supplement included glucose-enriched DMEM, glutamine-enriched DMEM, and the previous two groups each with sodium pyruvate added.

Supplemental Table 3: Cartridge layout of 3rd experiment. WUP (White DMEM, glucose, and sodium pyruvate) has 4.5g/L glucose, 110 mg/L sodium pyruvate, 25mM HEPES,10% FBS, and 1% PenStrep added. WNP (White DMEM, glutamine, and sodium pyruvate) has 0.7mM glutamine, 110 mg/L sodium pyruvate, 25mM HEPES,10% FBS, and 1% PenStrep added. WU (White DMEM, glucose, no sodium pyruvate) Has 4.5g/L glucose, 25mM HEPES,10% FBS, and 1% PenStrep added. WN (White DMEM, glutamine, no sodium pyruvate) Has 0.7mM glutamine, 25mM HEPES,10% FBS, and 1% PenStrep added. To ratio the oxygen and carbon sources correctly, media dilution was required. 2.5mL of media with 7.5mL of PBS yields approximately the correct ratio. From total capsule volume, 186uL will be media, and 264uL will be the air inside. Each gel has 150,000 OA cells embedded.

	**1**	**2**	**3**	**4**	**5**	**6**	**7**	**8**
**A**	WUPC1	WUPC2	WUC1	WUC2	WNPC1	WNPC2	WNC1	WNC2
**B**	WUP1	WUP2	WU1	WU2	WNP1	WNP2	WN1	WN2
**C**	WUP3	WUP4	WU3	WU4	WNP3	WNP4	WN3	WN4
**D**	WUP5	WUP6	WU5	WU6	WNP5	WNP6	WN5	WN6
**E**	WUP7	WUP8	WU7	WU8	WNP7	WNP8	WN7	WN8
**F**	WUPC3	WUPC4	WUC3	WUC4	WNPC3	WNPC4	WN3	WN4

Supplemental Table 4: Cartridge layout of final experiment. Groups were divided into either the No Cell (NC), Cell Only (CO), Cells + Glucose (Glu), or cells + Glutamine (Gln) groups. Details of this experimental design are in the body of the paper.

	Group 1		Group 2		Group 3		Group 4	
	Col 1	Col 2	Col 3	Col 4	Col 5	Col 6	Col 7	Col 8
Row A - References	NC C1	NC C2	CO C1	CO C2	Glu C1	Glu C2	Gln C1	Gln C2
Row B - Samples	NC 1	NC 2	CO 1	CO 2	Glu 1	Glu 2	Gln 1	Gln 2
Row C - Samples	NC 3	NC 4	CO 3	CO 4	Glu 3	Glu 4	Gln 3	Gln 4
Row D - Samples	NC 5	NC 5	CO 5	CO 6	Glu 5	Glu 6	Gln 5	Gln 6
Row E - Samples	NC 6	NC 7	CO 7	CO 8	Glu 7	Glu 8	Gln 7	Gln 8
Row F - References	NC C3	NC C4	CO C3	CO C4	Glu C3	Glu C4	Gln C3	Gln C4

Supplemental Table 5: Calculation spreadsheet used to determine correct media and air ratio for sample capsules.

**Donor to Acceptor Ratio**	**5.560184299**	
emols in headspace	8.750180015	emols
emols in 1x media	48.65261353	emols
Media Volume	186	uL
Agarose Volume	150	uL
Headspace Volume	320	uL
Total # of oxidizable emols/L 1x media	0.261573191	emols/L media
Electron acceptor capacity	0.027344313	emol capacity/L air
Ambient air pressure at location of experiment	0.83	atm
Experimental Temperature	310	K
Total capsule volume	656	uL
Degree of Reduction for O2	4	emols/mol O2
Molar concentration of O2 in air (L basis)	0.006836078	mol/L
Molar concentration of O2 in air (m^3 basis)	6.836078137	mol/m^3
Gas Constant	8.314	J/mol/K
Partial Pressure of O2	17618.89763	Pascals (N/m^2)
O2 fraction in atmosphere	0.2095	
Air pressure	84099.75	Pascals (N/m^2)

**README!**	
Purple (Final Result)	The dilution of 1x media that should be used to make the electron donor/acceptor ratio equal to 1
Blue (Input Conditions)	Experimental information that you need to input, will change based on the conditions of your experiment
Green (Part of Final Formula, Don't Change)	Formulas/arrays that directly contribute to the final volume value
Red (Don't Change)	Formulas/values that should typically be left alone

Supplemental Equation 1: Calculation of Lost Heat Generation from Cartilage Thinning and Chondrocyte Cell Count Reduction

Given that heat generation is dependent on cell count per unit area (C_A, [cells/mm^2]), heat generation per cell (Q_{Cell}, [J/cell]), and thickness of the articular cartilage (t, [mm]), heat generation per unit depth (Q_{total}, [J/mm]) is described by:

$$Q_{total} = C_A Q_{Cell} t$$

For a healthy cell count of C_A = 4,636 cells/mm^2 (*Danalache et al)*, a heat generation value of Q_{Cell} = 2.79 μJ/cell gathered through our experimentation for a healthy cell group with access to glucose as a main carbon source, and a healthy articular cartilage thickness of t = 3.9mm, this yields a heat generation per unit depth of 50.4mJ/mm.

For a decreased cell count of C_A = 1,622 cells/mm^2 (*Danalache et al),* the same heat generation value of Q_{Cell} = 2.79 μJ/cell, and a reduced cartilage thickness of t = 3.68mm, this yields a decreased heat generation per unit thickness of 16.67mJ/mm, an overall decrease of 67% of the heat generated by thicker, more cell dense cartilage.

Supplemental Material 1: Statistical Report on Total Main Heat

2024-10-23

Data Wrangling

In the data wrangling process, the columns “Sample Group” and “Total main heat” were renamed to SampleGroup and TotalHeat. SampleGroup was converted to a factor and re-coded to levels: Glu, Gln, CO, and NC. The measurement column was split into two new factor variables, row and column, by extracting the first and second characters, respectively. Additionally, a new column was created to store the log-transformed values of TotalHeat.

```
#read in the data
df_cell <- read_csv(file = "(4) No Cells, Cells,Glu,Gln 06_24_Total_Heat.csv"
,
                    locale = locale(encoding = "latin1"))

# data wrangling
df_cell2 <- df_cell %>% dplyr::rename(SampleGroup = 'Sample Group',
                                   TotalHeat = 'Total main heat (µJ)') %>%
      mutate(SampleGroup = factor(SampleGroup),
             SampleGroup = fct_recode(SampleGroup, 'Glu' = 'Cells + Glucose (
Glu)',
                                      'Gln' = 'Cells + Glutamine (Gln)',
                                      'CO' = 'Cells Only (CO)',
                                      'NC' = 'No Cell (NC)'),
             Row = factor(substr(Measurement, start = 1, stop = 1)),
             Column = factor(substr(Measurement, start = 2, stop = 2))
             )

df_cell2 <- df_cell2 %>% mutate(LogTotalHeat = log(TotalHeat)) #Add logheat t
o data set


levels(df_cell2$SampleGroup)

## [1] "Glu" "Gln" "CO"  "NC"

df_cell2 <- df_cell2 %>% mutate(Sample_Group =
                                  factor(SampleGroup, levels = c("NC", "CO",
"Glu", "Gln")))
#Reorder: NC CellsOnly, Glucose, Glutamine



#get summary statistics
favstats(TotalHeat ~ Sample_Group, data = df_cell2)
```

```
##   Sample_Group        min        Q1    median        Q3       max       mea
n
## 1           NC 0.03255085 0.2316118 0.2822333 0.5859425 0.7419047 0.374019
4
## 2           CO 1.99149639 2.8161522 2.9986614 3.3053231 3.9727835 3.033071
6
## 3          Glu 1.53895717 2.4758575 2.8164107 3.0466807 4.3352869 2.791378
5
## 4          Gln 0.68079853 1.6860614 1.9461031 2.2679942 2.8268506 1.900349
8
##          sd n missing
## 1 0.2511562 8       0
## 2 0.5737868 8       0
## 3 0.8191088 8       0
## 4 0.6502120 8       0
```

```
# Colors to use:

#No Cells (Grey): #6E6E6E
#Cells Only (Black): #000000
#Cells + Glucose (Red): #8B0000
#Cells + Glutamine (Blue): #104E8B

palette_new <- grDevices::colorRampPalette(
          colors = c("#6E6E6E", "#000000", "#8B0000", "#104E8B"))(4)
```

Raw Data Visualizations

After data wrangling, we proceeded to visualize the raw data. The figures show the total main heat generated by four sample groups: Cells + Glucose (Glu), Cells + Glutamine (Gln), Cells Only (CO), and No Cell (NC). The distribution of each group is visualized, with black dots representing the group means and vertical lines representing 95% confidence intervals.

```
# visualize the raw data
Rawdatabygroups <- enhanced_stripchart(TotalHeat ~ Sample_Group, data = df_ce
ll2, ptalpha = 1) +
  labs(title = "Enhanced Stripchart of Total Main Heat Generated by the Sampl
es",
       y = "Total Main Heat",
       x = "Sample Group")+
  theme(plot.title = element_text(face = "bold", hjust = 0.5, size = 15),
        axis.title = element_text(size = 15),
        axis.text = element_text(size= 15)) +
  scale_colour_manual(values = palette_new)


#Change color, point alpha

Rawdatabygroups
```

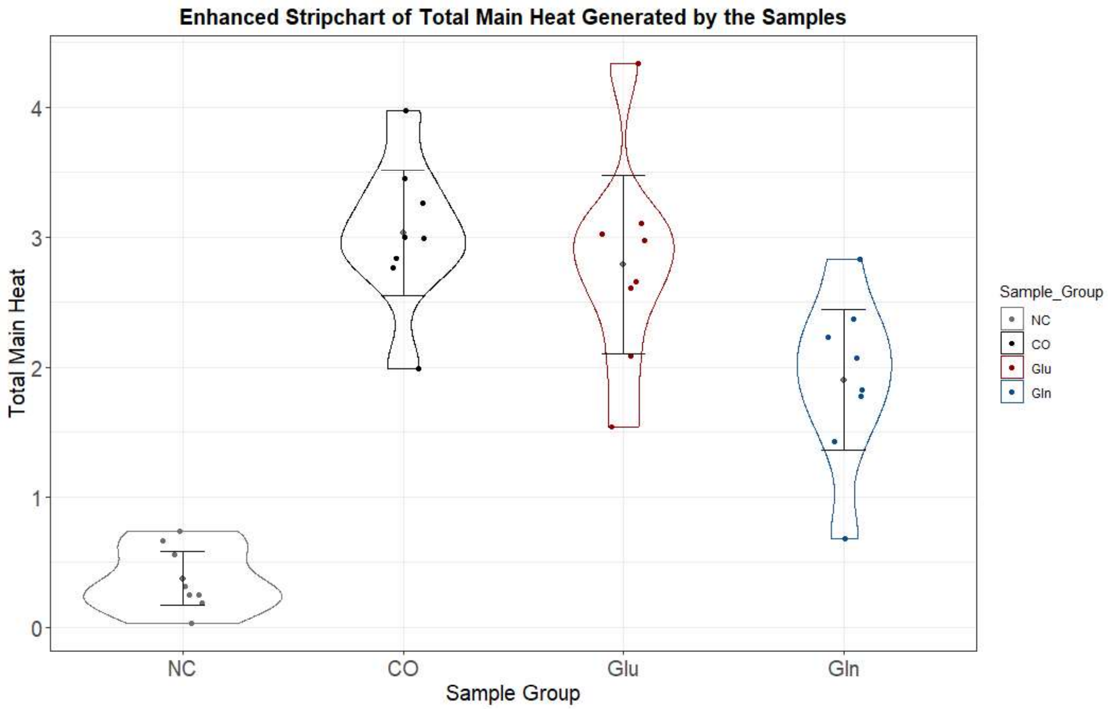

Enhanced Stripchart of the total main heat generated by the sample groups

```
ggsave(plot = Rawdatabygroups, "Rawdatabygroups.pdf", width = 12, height = 8)

# visualize the raw data
enhanced_stripchart(LogTotalHeat ~ Sample_Group, data = df_cell2) +
  labs(title = "Enhanced Stripchart of Log Total Main Heat Generated by the S
amples",
       y = "Log Total Main Heat",
       x = "Sample Group")+
  theme(plot.title = element_text(face = "bold", hjust = 0.5, size = 15),
        axis.title = element_text(size = 15),
        axis.text = element_text(size= 15)) +
  scale_colour_manual(values = palette_new)
```

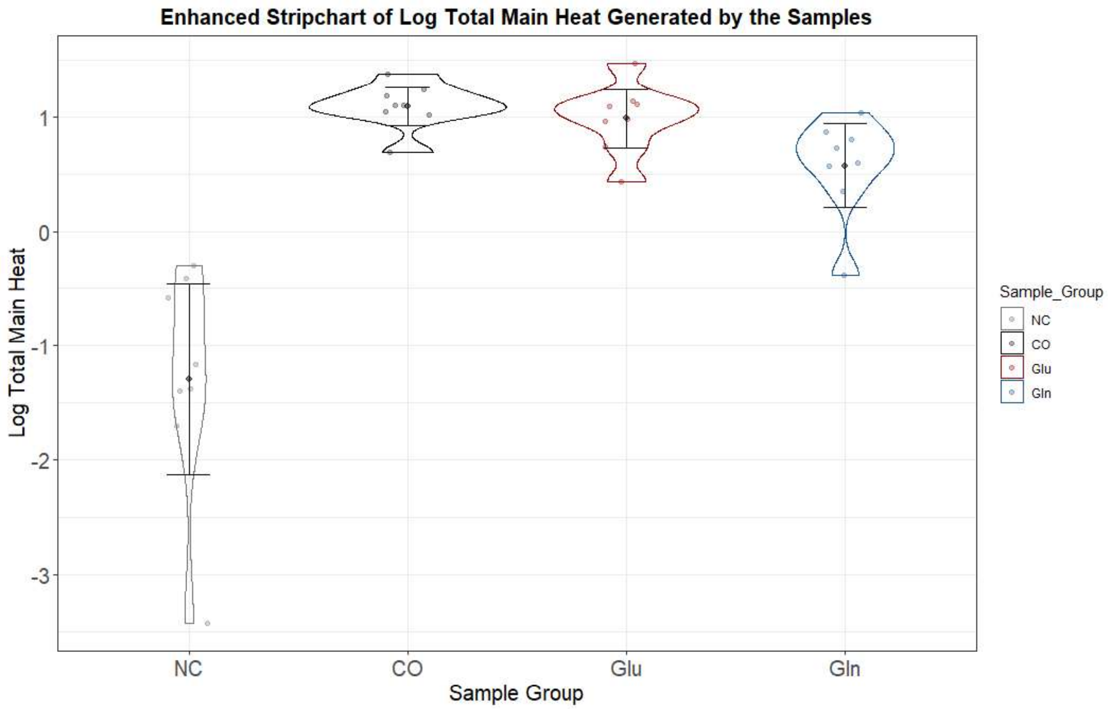

Enhanced Stripchart of the log-total main heat generated by the sample groups

- This suggests that the log-transformation is not going to be successful in stabilizing the variance across the groups…

```
# visualize the raw data
Rawdatabyrows <- enhanced_stripchart(TotalHeat ~ Row, data = df_cell2) +
  labs(title = "Enhanced Stripchart of Total Main Heat Generated by the row",
       y = "Total Main Heat",
       x = "Row")+
  theme(plot.title = element_text(face = "bold", hjust = 0.5, size = 15),
        axis.title = element_text(size = 15),
        axis.text = element_text(size= 15))

Rawdatabyrows
```

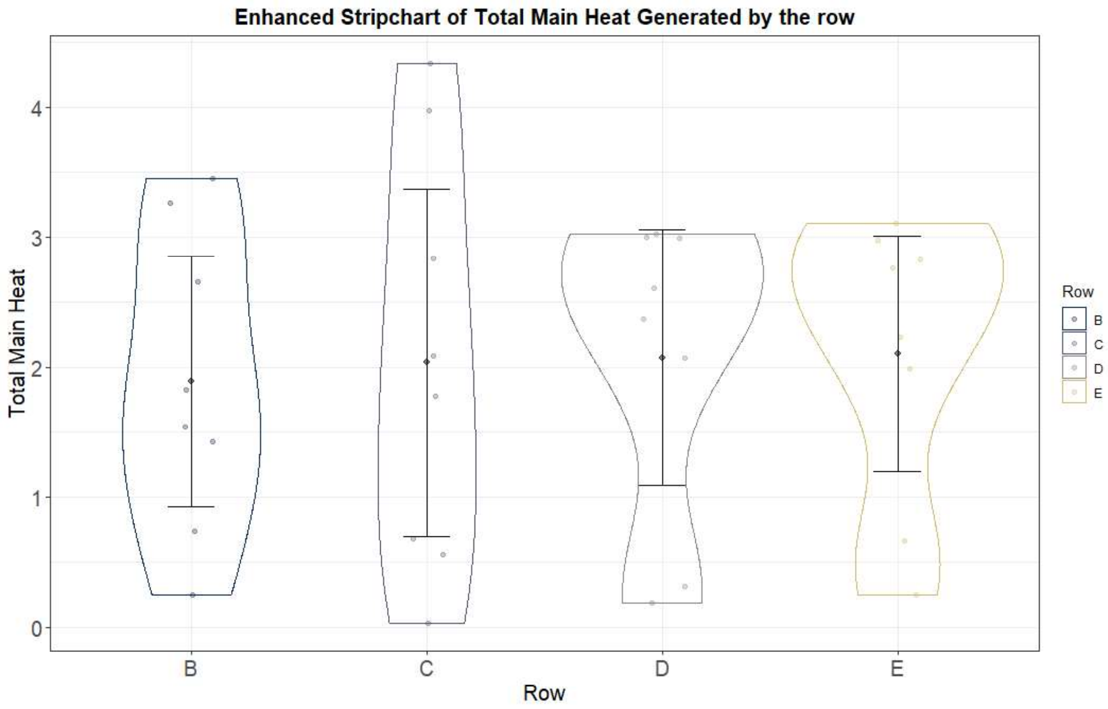

Enhanced Stripchart of the total main heat generated by row

```
ggsave(plot = Rawdatabyrows, "Rawdatabyrows.pdf", width = 12, height = 8)

# visualize the raw data
Rawdatabycolumns <- enhanced_stripchart(TotalHeat ~ Column, data = df_cell2)
+
  labs(title = "Enhanced Stripchart of Total Main Heat Generated by column",
       y = "Total Main Heat",
       x = "Column")+
  theme(plot.title = element_text(face = "bold", hjust = 0.5, size = 15),
        axis.title = element_text(size = 15),
        axis.text = element_text(size= 15))
Rawdatabycolumns
```

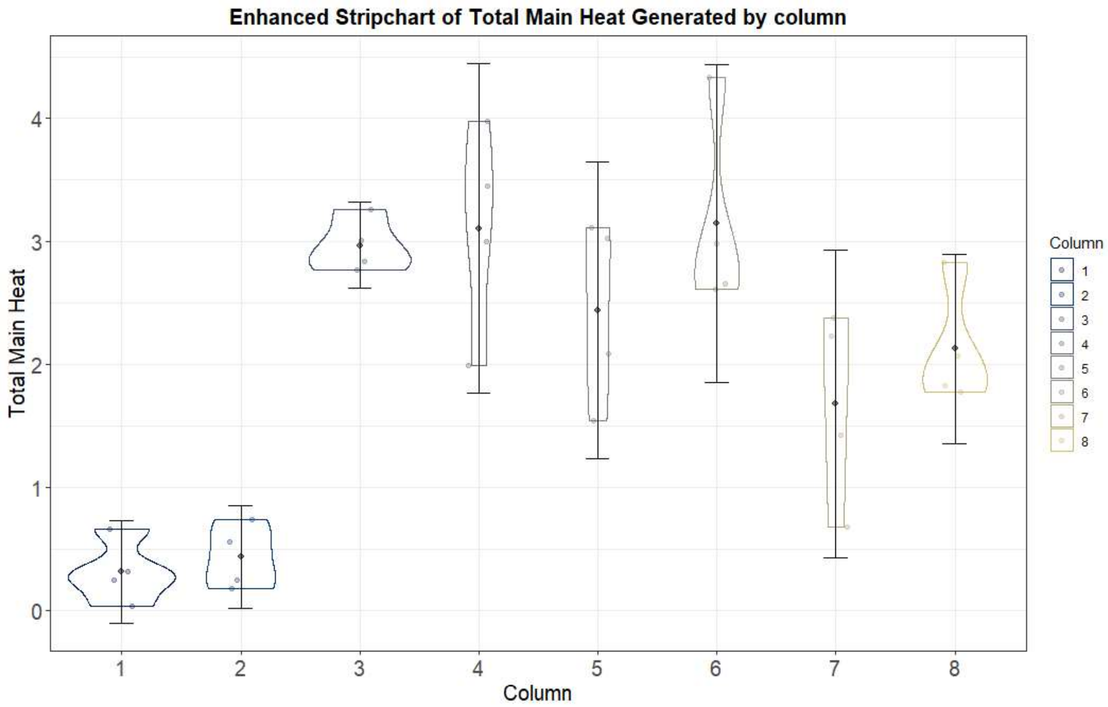

Enhanced Stripchart of the total main heat generated by columns

```
ggsave(plot = Rawdatabycolumns, "Rawdatabycolumns.pdf", width = 12, height =
8)
```

Linear Model

First, we fit an ANOVA model. This model assumes that each of the samples is independent, which isn't entirely true for these data because we have repeated measurements across rows and columns (samples organized down the column and samples also organized across each row). The columns could be more impactful on the measurements, but each pair of neighboring columns is assigned the same treatment levels. This model also assumes that the variability of the residuals is consistent across the groups, which we assess by looking at a Residual vs Fitted plot. The model also assumes that the residuals follow a normal distribution, which we assess using a normal QQ plot of the residuals.

```
# fit a linear model
a_model <- lm(TotalHeat ~ Sample_Group, data = df_cell2)
Anova(a_model)

## Anova Table (Type II tests)
##
## Response: TotalHeat
##               Sum Sq Df F value    Pr(>F)
## Sample_Group 34.759  3  31.187 4.544e-09
## Residuals    10.402 28

# diagnostic plots
resid_panel(a_model, "R")
```

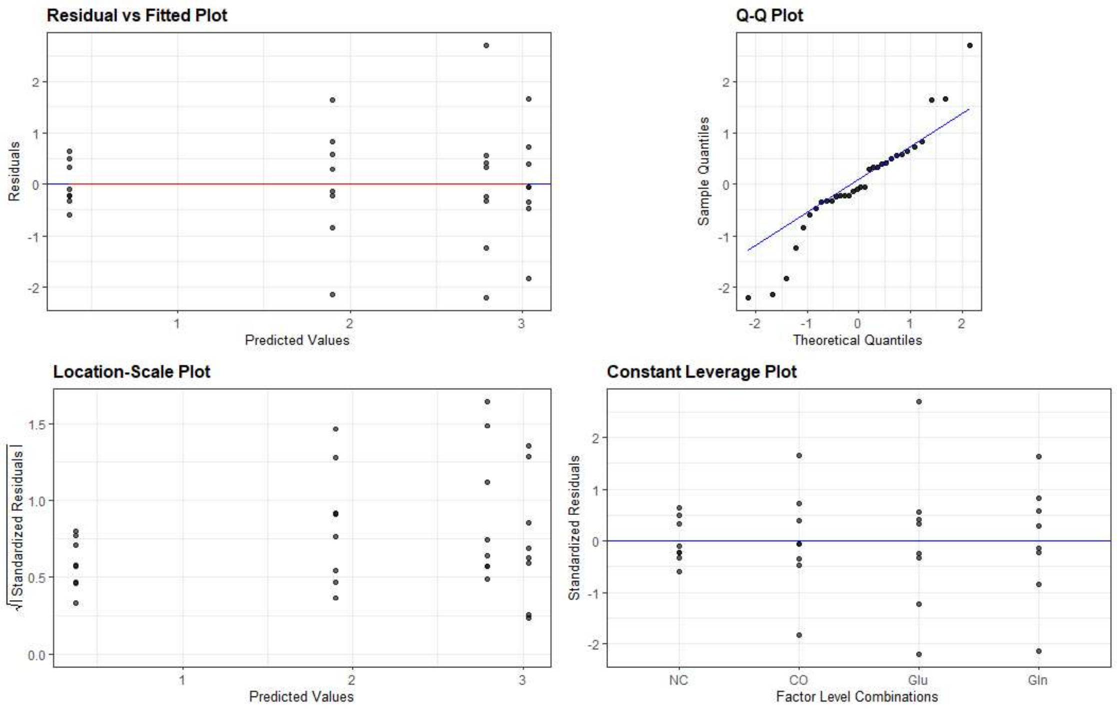

- There is a clear issue with non-constant variance and some heavy-tailed aspects to the residual distribution, suggesting an issue with the normality of residuals assumption.
- This model does not account for the row-to-row or column-to-column variation except through the treatment levels that are applied to neighboring pairs of columns.

```
# visualize the results of the ANOVA model
plot(allEffects(a_model), grid = T)
```

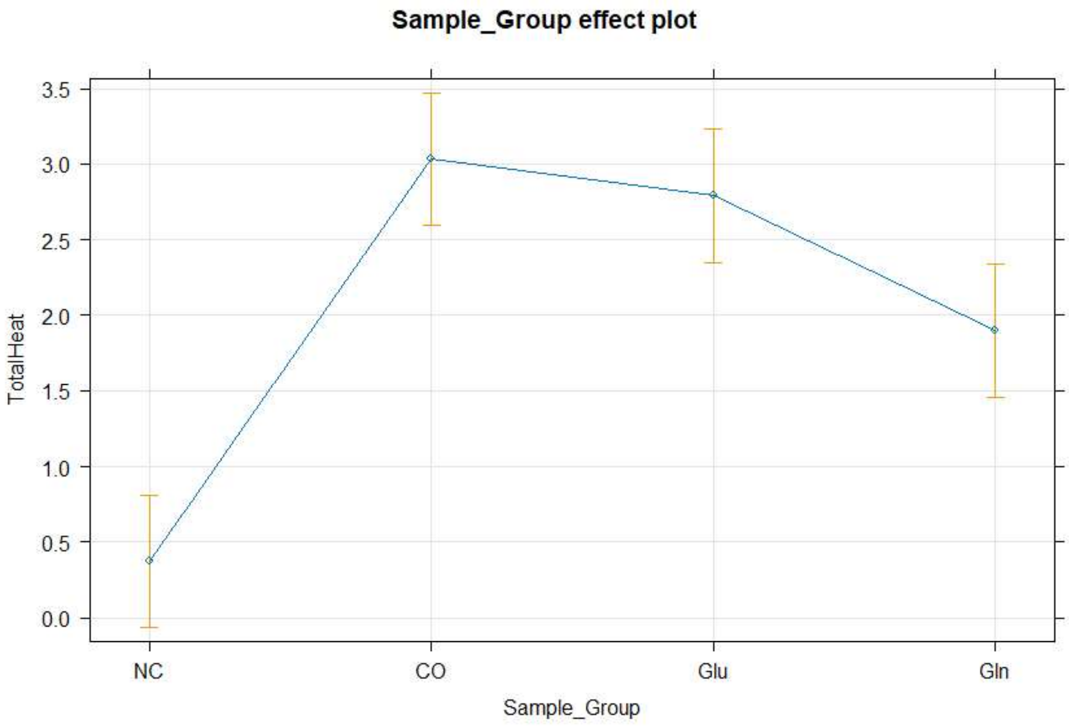

Plot of the estimated group means of the total main heat from the ANOVA model along with 95% confidence intervals.

Non-constant variance models for TotalHeat:

- Welch's F-test

Since the residual diagnostics indicated non-constant variance, we performed Welch's F-Test, which adjusts for unequal variances between groups and is better suited for situations where the assumption of equal variance is violated.

```
# Perform Welch's F-Test
welch_test <- welch.test(TotalHeat ~ Sample_Group, data = df_cell2)

##
##   Welch's Heteroscedastic F Test (alpha = 0.05)
## -------------------------------------------------------------
##   data : TotalHeat and Sample_Group
##
##   statistic  : 61.84811
##   num df     : 3
##   denom df   : 13.89615
##   p.value    : 2.778116e-08
##
##   Result     : Difference is statistically significant.
## -------------------------------------------------------------
```

- It is also possible to use a function that can handle more complex situations that gives a similar model with different variances estimated for each group, using `gls` and variances changing based on groups of the predictor.

Given the non-constant variance across sample groups, we fit a Generalized Least Squares (GLS) model that allows for different variances based on groups of the predictor.

```
# Fit the GLS model with different variances for each group
gls_model <- gls(TotalHeat ~ Sample_Group, data = df_cell2,
                 weights = varIdent(form = ~ 1 | Sample_Group))
summary(gls_model)

## Generalized least squares fit by REML
##   Model: TotalHeat ~ Sample_Group
##   Data: df_cell2
##       AIC      BIC    logLik
##   67.8379 78.49554 -25.91895
##
## Variance function:
##  Structure: Different standard deviations per stratum
##  Formula: ~1 | Sample_Group
##  Parameter estimates:
##       NC       CO      Glu      Gln
## 1.000000 2.284581 3.261353 2.588875
##
## Coefficients:
##                     Value  Std.Error   t-value p-value
## (Intercept)     0.3740194 0.08879712  4.212067   2e-04
## Sample_GroupCO  2.6590522 0.22144713 12.007616   0e+00
## Sample_GroupGlu 2.4173591 0.30290650  7.980546   0e+00
## Sample_GroupGln 1.5263304 0.24643840  6.193557   0e+00
##
##  Correlation:
##                 (Intr) Sm_GCO Sampl_GrpGl
## Sample_GroupCO  -0.401
## Sample_GroupGlu -0.293  0.118
## Sample_GroupGln -0.360  0.144  0.106
##
## Standardized residuals:
##         Min          Q1         Med          Q3         Max
## -1.87562096 -0.49107967 -0.09186096  0.56371174  1.88486345
##
## Residual standard error: 0.2511562
## Degrees of freedom: 32 total; 28 residual

# Residuals vs Fitted plot
plot(gls_model, form = resid(., type = "p") ~ fitted(.))
```

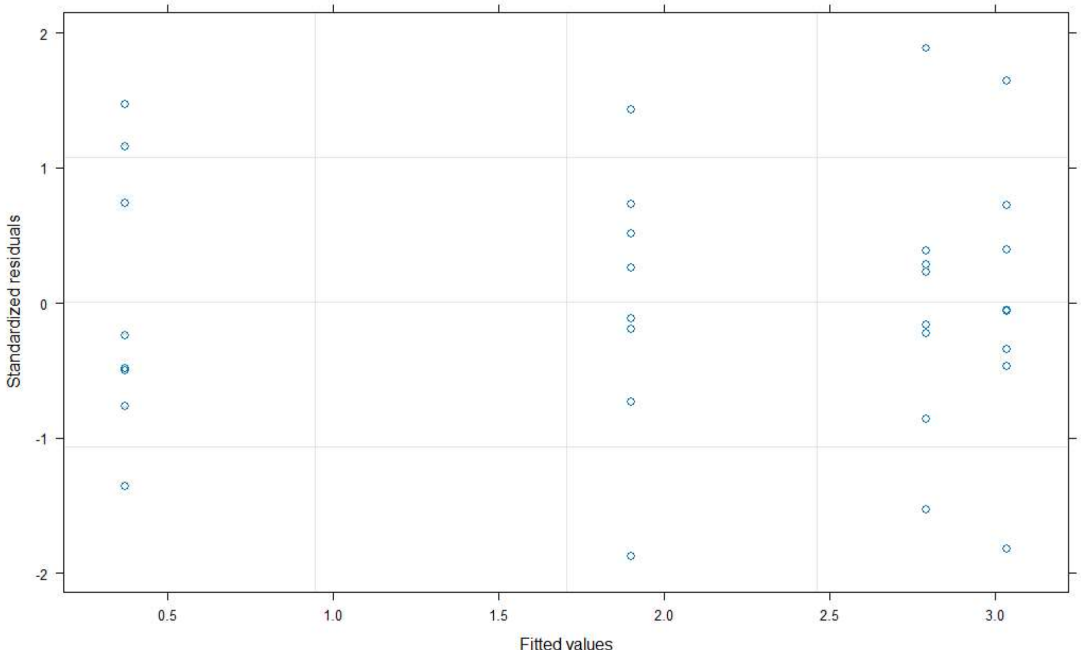

- Now there is little to no evidence of a violation of the equal variance assumption since the vertical spread of the residuals after accounting for different variances is consistent across the groups.

```r
# Q-Q Plot for Residuals
qqnorm(resid(gls_model, type = "p"))
qqline(resid(gls_model, type = "p"))
```

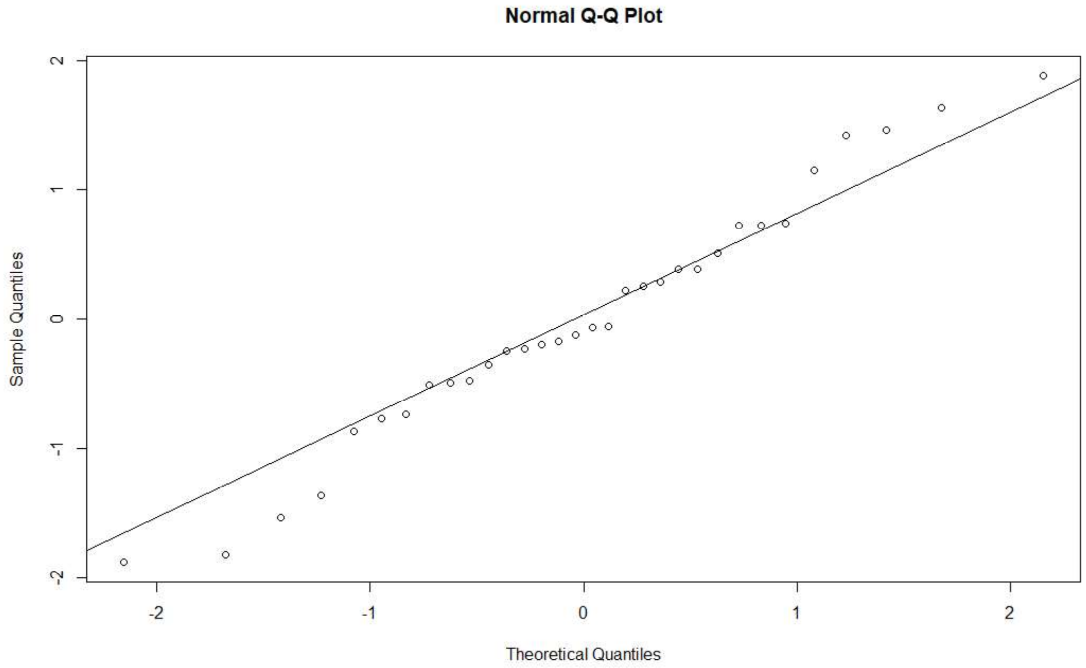

- There is less of an issue with, but still some, heavy-tailed aspects to the residual distribution.

Test for group differences

```
anova(gls_model)

## Denom. DF: 28
##               numDF   F-value p-value
## (Intercept)       1 199.17546  <.0001
## Sample_Group      3  67.78242  <.0001

welch_test <- welch.test(TotalHeat ~ Sample_Group, data = df_cell2)

##
##   Welch's Heteroscedastic F Test (alpha = 0.05)
## -------------------------------------------------------------
##   data : TotalHeat and Sample_Group
##
##   statistic  : 61.84811
##   num df     : 3
##   denom df   : 13.89615
##   p.value    : 2.778116e-08
##
##   Result     : Difference is statistically significant.
## -------------------------------------------------------------
```

- There is very strong evidence against the null hypothesis that the true mean total main heat is the same for all groups ($F(3, 28) = 67.8$, p-value < 0.0001), so we can conclude that at least one sample group is different from the others.

```
# visualize the results of the ANOVA model
plot(allEffects(gls_model), grid = T)
```

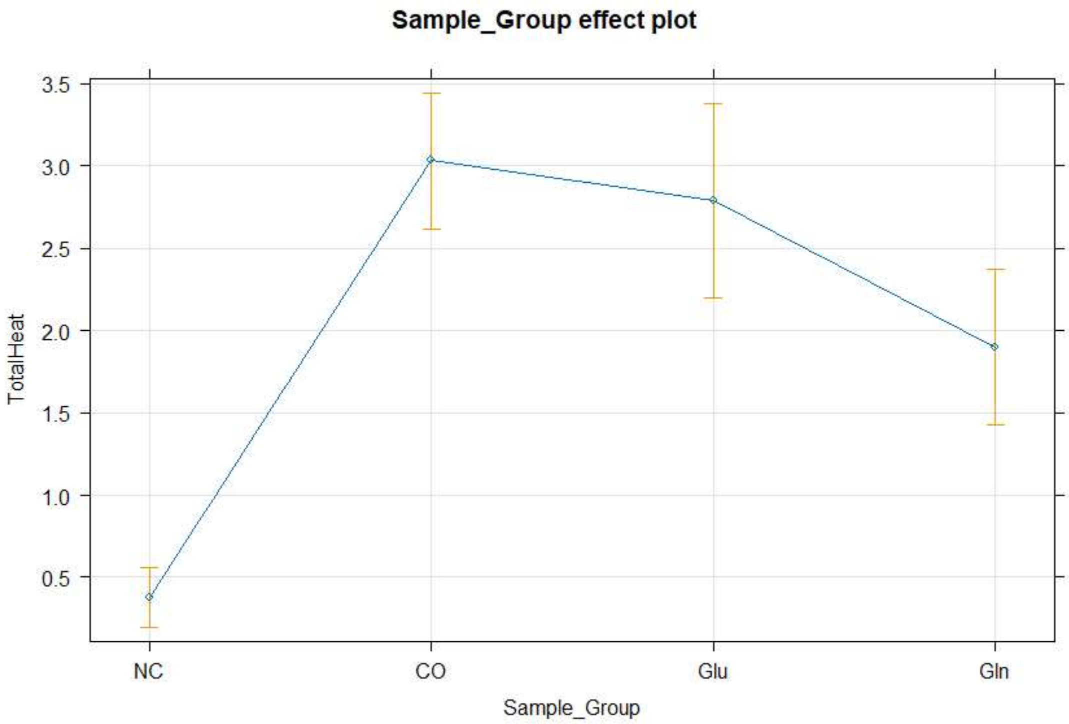

Plot of the estimated group means of the total main heat from the non constant variance model along with 95% confidence intervals.

Multiple Comparison Test

The results above showed evidence of difference in the mean total main heat for at least one sample group. We conducted a follow up test using the Tukey's Honest Significant Difference (HSD) test to identify which specific groups differ in mean total heat.

```
# Perform pairwise comparisons using emmeans with Tukey adjustment
gls_emmeans <- emmeans(gls_model, pairwise ~ Sample_Group, adjust = "tukey")

emmeans_results <- cld(gls_emmeans$emmeans,Letters = letters)
emmeans_results

##  Sample_Group emmean     SE df lower.CL upper.CL .group
##  NC            0.374 0.0888  7    0.164    0.584  a
##  Gln           1.900 0.2299  7    1.357    2.444   b
##  Glu           2.791 0.2896  7    2.107    3.476   bc
##  CO            3.033 0.2029  7    2.553    3.513    c
##
## Degrees-of-freedom method: satterthwaite
## Confidence level used: 0.95
## P value adjustment: tukey method for comparing a family of 4 estimates
## significance level used: alpha = 0.05
## NOTE: If two or more means share the same grouping symbol,
```

```
##       then we cannot show them to be different.
##       But we also did not show them to be the same.

Pairwisegls <- plot(gls_emmeans, comparison = T)

Pairwisegls
```

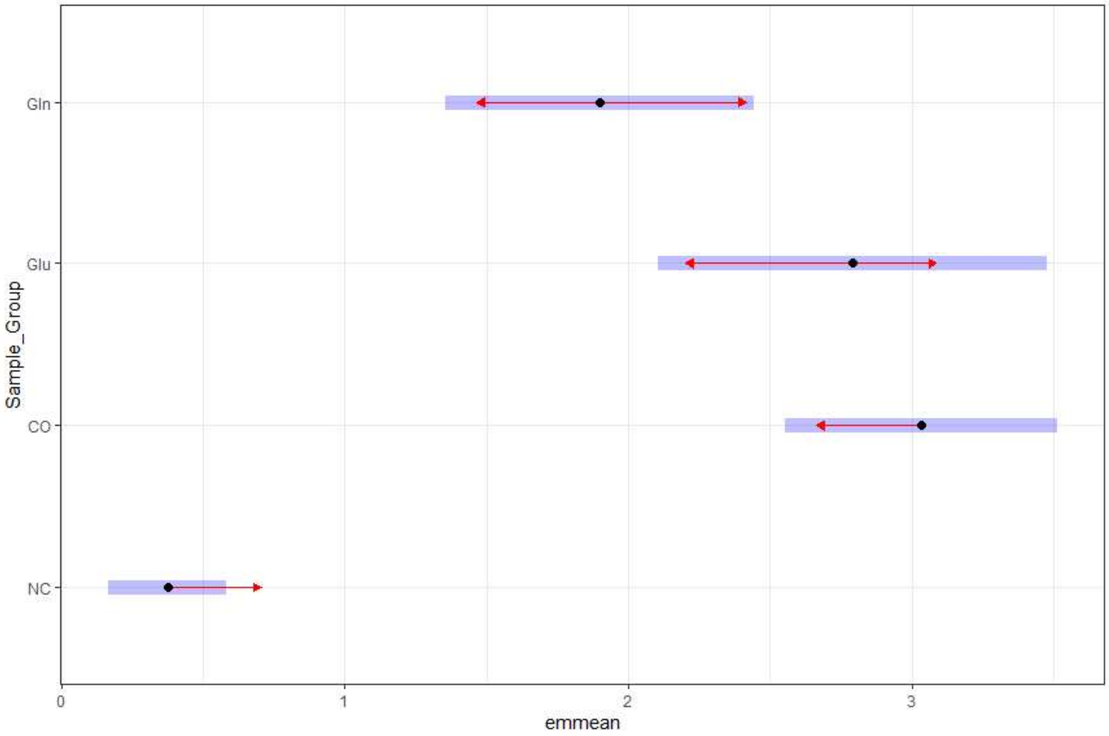


```
ggsave(plot = Pairwisegls, "Pairwisegls.pdf", width = 12, height = 8)
```

- Red arrows and their overlap is about the test for differences at the 5% familywise significance level, the blue bars are the individual group mean 95% CIs.
- There is insufficient evidence to show that these pair of groups are different.
  - Gln - Glu
  - Glu - CO
- However, there is enough evidence to show that all other group pairs are different.

Mixed Model

Now, we fit a mixed effects model that addresses the non-independence in the total main heat generated by the same sample. If only one random effect can be considered due to the columns sharing information with the grouping variable, accounting just for row-to-row variation seems worth trying. The mixed effects model assumes that the vertical spread of the residuals is consistent across the sample groups. The model also assumes that both the residuals and the random effects follow a normal distribution.

The theoretical model is given by

$$y_{ij} = \mu_i + Row_i + \varepsilon_{ij},$$

where

$$\mu_i = \beta_0 + \beta_1 I_{(SG=CO)} + \beta_2 I_{(SG=Glu)} + \beta_3 I_{(SG=Gln)}$$

,

$$\varepsilon_{ij} \sim N(0, \sigma_y^2)$$

$$Row_i \sim N(0, \sigma_{Row}^2)$$

,

$i = 1, \cdots, 4$ $j = 1, .., 8$ for the column "within" each row of the observations.

Row_i - random effect associated with the i-th row.

Just Row random effects:

```
lme_model <- lme(TotalHeat ~ Sample_Group, random = ~1 | Row, data = df_cell2
,
                 weights = varIdent(form = ~ 1 | Sample_Group))
summary(lme_model)

## Linear mixed-effects model fit by REML
##   Data: df_cell2
##        AIC      BIC    logLik
##   69.8379 81.82774 -25.91895
##
## Random effects:
##  Formula: ~1 | Row
##          (Intercept)  Residual
## StdDev: 7.884697e-06 0.2511562
##
## Variance function:
##  Structure: Different standard deviations per stratum
##  Formula: ~1 | Sample_Group
##  Parameter estimates:
##       NC       CO      Glu      Gln
## 1.000000 2.284580 3.261352 2.588875
## Fixed effects:  TotalHeat ~ Sample_Group
##                     Value  Std.Error DF   t-value p-value
## (Intercept)     0.3740194 0.08879714 25  4.212066   3e-04
## Sample_GroupCO  2.6590522 0.22144709 25 12.007618   0e+00
## Sample_GroupGlu 2.4173591 0.30290652 25  7.980545   0e+00
## Sample_GroupGln 1.5263304 0.24643841 25  6.193557   0e+00
```

```
##  Correlation: 
##                  (Intr) Sm_GCO Sampl_GrpGl
## Sample_GroupCO  -0.401                    
## Sample_GroupGlu -0.293  0.118             
## Sample_GroupGln -0.360  0.144  0.106      
## 
## Standardized Within-Group Residuals:
##         Min          Q1         Med          Q3         Max 
## -1.87562092 -0.49107957 -0.09186097  0.56371178  1.88486337 
## 
## Number of Observations: 32
## Number of Groups: 4
```

- The results of the mixed-effects model show that the variance for the row random effects is very close to 0, indicating little to no variation between the rows. As a result, including random effects may not be necessary in this model.

```r
plot(lme_model, form = resid(., type = "p") ~ fitted(.))
```

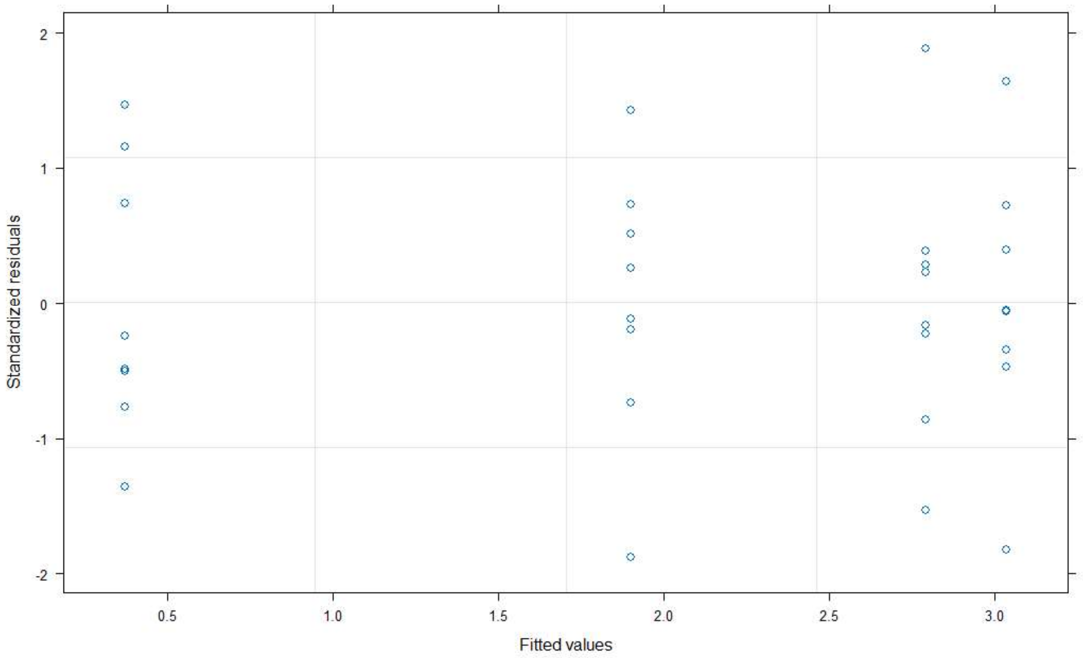


```r
qqnorm(resid(lme_model, type = "p"))
qqline(resid(lme_model, type = "p"))
```

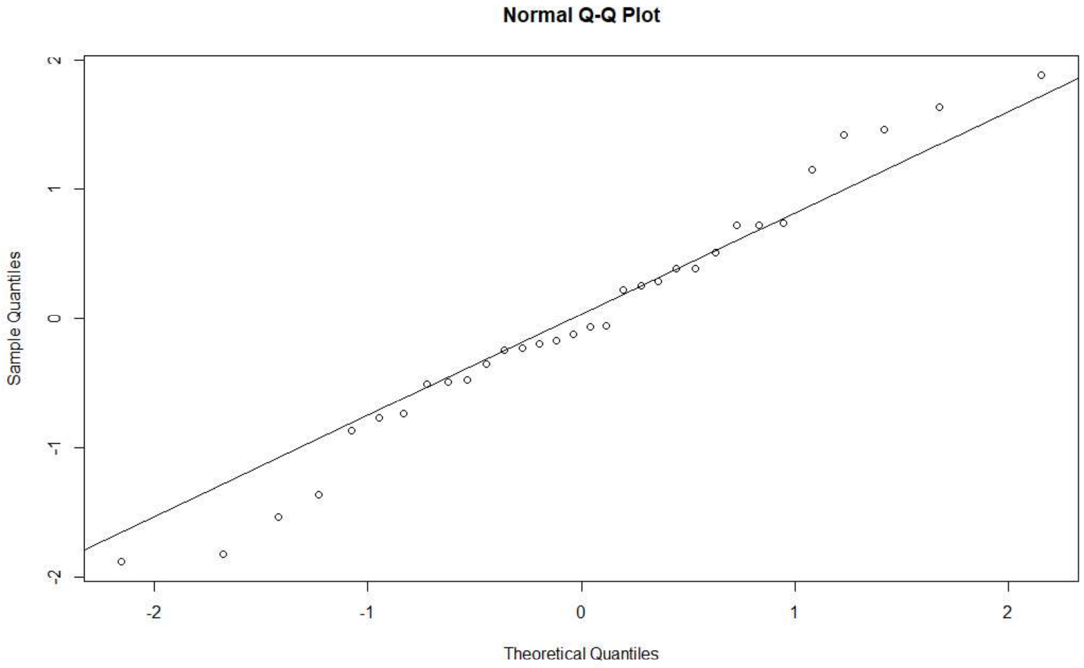

- There are no clear issues with non-constant variance, though the residual distribution shows some heavy-tailed aspects.

```
anova(lme_model)

##               numDF denDF   F-value p-value
## (Intercept)       1    25 199.17553  <.0001
## Sample_Group      3    25  67.78243  <.0001

# Anova(lme_model)
```

- The row random effect may not have impacted the model but it reduces the denominator DF for the F-statistic from 28 to 25.

```
# visualize the results of the ANOVA model
plot(allEffects(lme_model), grid = T)
```

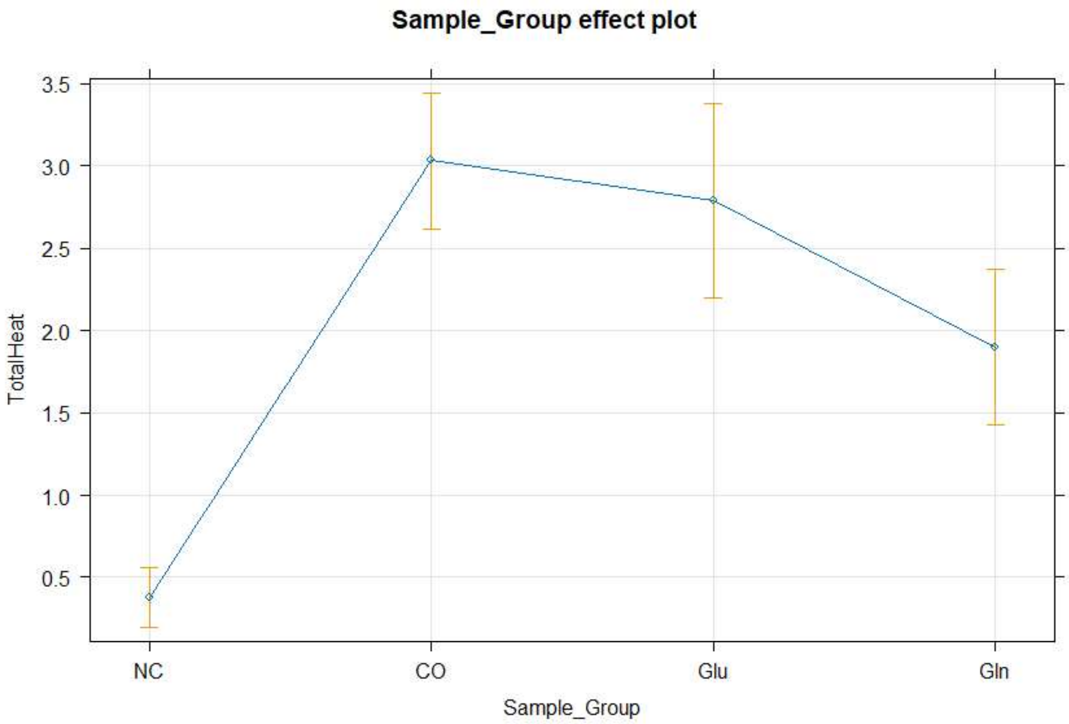

Plot of the estimated group means of the total main heat from the mixed effect model with row random effect and non constant variance along with 95% confidence intervals.

Pairwise comparison, non-constant variance, row random effect

```
lme_emmeans <- emmeans(lme_model, pairwise ~ Sample_Group, adjust = "tukey")
lme_results <- cld(lme_emmeans$emmeans,Letters = letters)
lme_results

##  Sample_Group emmean     SE df lower.CL upper.CL .group
##  NC            0.374 0.0888  3   0.0914    0.657  a
##  Gln           1.900 0.2299  3   1.1688    2.632   b
##  Glu           2.791 0.2896  3   1.8697    3.713   bc
##  CO            3.033 0.2029  3   2.3875    3.679    c
##
## Degrees-of-freedom method: containment
## Confidence level used: 0.95
## P value adjustment: tukey method for comparing a family of 4 estimates
## significance level used: alpha = 0.05
## NOTE: If two or more means share the same grouping symbol,
##       then we cannot show them to be different.
##       But we also did not show them to be the same.

plot(lme_emmeans, comparison = TRUE)
```

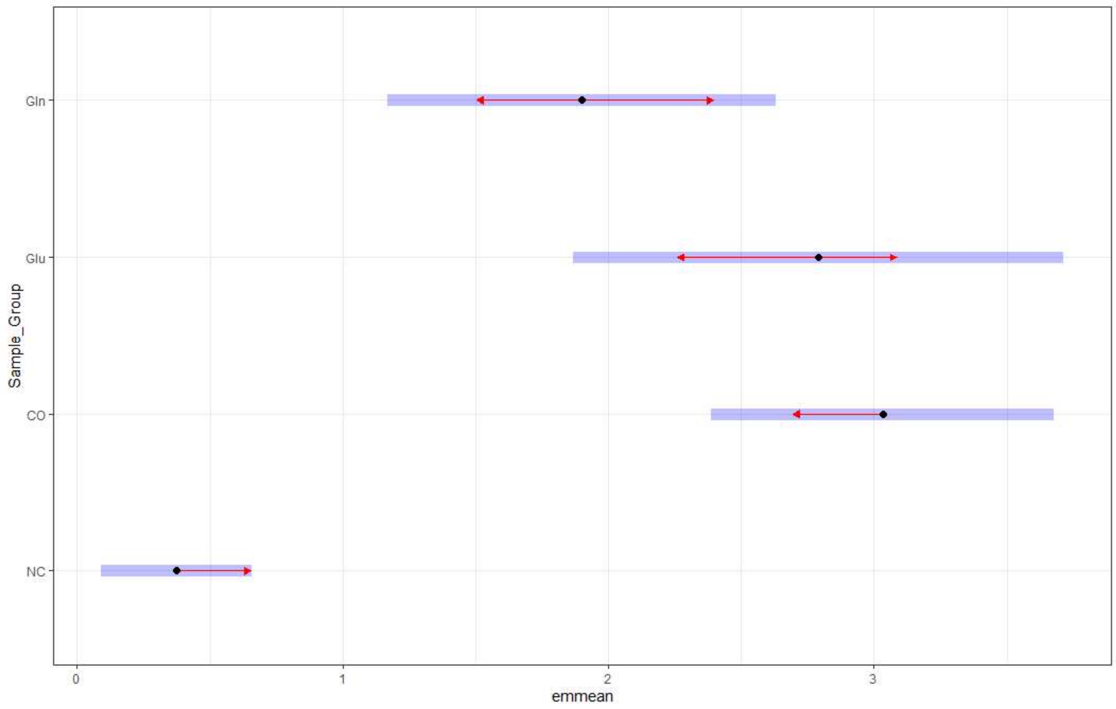

- There is not enough evidence to show that these groups are different.
- Gln - Glu
- Glu - CO
- There is evidence to show that all other pairs are different.
- These results are consistent with those from the non-constant variance model.

Evidence for differing variances and comparing variances:

- It is possible to do a likelihood ratio test that assesses evidence against the null hypothesis of $H_0: \sigma^2_{NC} = \sigma^2_{CO} = \sigma^2_{GLn} = \sigma^2_{Glu}$, that all the groups have the same variances for either the models with or without the row random effects. Results are discussed just for the model without the row random effect.
- For the model without the row random effect:

```
gls_modelHOV <- gls(TotalHeat ~ Sample_Group, data = df_cell2, method = "REML
")
anova(gls_modelHOV, gls_model)

##              Model df      AIC      BIC    logLik   Test  L.Ratio p-value
## gls_modelHOV     1  5 70.05303 76.71405 -30.02651
## gls_model        2  8 67.83790 78.49554 -25.91895 1 vs 2 8.215123  0.0418
```

- In the model without a row random effect, there is moderate evidence for differing variances in the groups with $\chi^2_3 = 8.2$ and p-value = 0.042. In this model, the estimated variance for the “no cells” group but in the same row is $\hat{\sigma}^2_{error} =$

$0.25116^2 = 0.0631$. The model estimated variance for CO is $0.25116^2 \times 2.285^2 = 0.329$, for Glu it is $0.25116^2 \times 3.26^2 = 0.671$, and for Gln it is $0.25116^2 \times 2.589^2 = 0.423$

- Note that this model is simple enough that those results are basically the same as using estimated variances for each of the groups from the original data set, but adjusting the inferences for the means for these differences:

```
var(TotalHeat ~ Sample_Group, data = df_cell2)

##         NC         CO        Glu        Gln
## 0.06307943 0.32923125 0.67093930 0.42277561
```

**Donor to Acceptor Ratio**	**5.560184299**
emols in headspace	8.750180015
emols in 1x media	48.65261353
Media Volume	186
Agarose Volume	150
Headspace Volume	320
Total # of oxidizable emols/L 1x media	0.261573191
Electron acceptor capacity	0.027344313
Ambient air pressure at location of experiment	0.83
Experimental Temperature	310
Total capsule volume	656
Degree of Reduction for O2	4
Molar concentration of O2 in air (L basis)	0.006836078
Molar concentration of O2 in air (m^3 basis)	6.836078137
Gas Constant	8.314
Partial Pressure of O2	17618.89763
O2 fraction in atmosphere	0.2095
Air pressure	84099.75

README!

Purple (Final Result)	The dilution of 1x media that
Blue (Input Conditions)	Experimental information tha
Green (Part of Final Formula, Don't Change)	Formulas/arrays that directly
Red (Don't Change)	Formulas/values that should t

Units
emols
emols
uL
uL
uL
emols/L media
emol capacity/L air
atm
K
uL
emols/mol O2
mol/L
mol/m^3
J/mol/K
Pascals (N/m^2)
Pascals (N/m^2)

should be used to make the electron donor/acceptor ratio equal to 1
t you need to input, will change based on the conditions of your experiment
contribute to the final volume value
typically be left alone

	Mass per volume for each component (g/L)
Glucose	1.125
Pyruvate	0.0275
L-Arginine HCl	0.021
Glycine	0.0075
L-Histidine HCl	0.0105
L-Isoleucine	0.02625
L-Leucine	0.02625
L-Lysine HCl	0.365
L-Phenylalanin	0.0165
L-Serine	0.0105
L-Threonine	0.02375
L-Tyrosine	0.0300925
L-Valine	0.0235
Adenine	0
Uracil	0
Cytosine	0
Guanine	0
Glucose	0
Sodium Citrate	0
L-Alanine	0
L-Asparagine	0
L-Aspartic Acid	0
L-Glutamic Aci	0
L-Proline	0
L-Cystine	0
L-Methionine	0
L-Tryptophan	0

MW (g/mol) of each component	# of carbon moles in each component
180	6
88.06	3
211	6
75	2
210	6
131	6
131	6
183	6
165	9
105	3
119	4
181	9
117	5
135	5
112	4
111	4
151	5
180	6
258	6
89	3
132	4
133	4
147	5
115	5
240	
149	
204	11

Degree of reduction for each component
4
3.33
5.67
4.5
4.83
5.5
5.5
5.67
4.78
4.33
4.75
4.56
5.4
5
4
4.75
4.6
4
3
5
4.5
3.75
4.2
5
4.73

Molar concentration of organic components
0.00625
0.000312287
9.95261E-05
0.0001
0.00005
0.000200382
0.000200382
0.001994536
0.0001
0.0001
0.00019958
0.000166257
0.000200855
0
0
0
0
0
0
0
0
0
0
0
0
0
0
0
0
0
0
0
0
0
0
0
0
0
0

**Donor to Acceptor Ratio**	**2.626758934**
emols in headspace	8.750180015
emols in 1x media	22.98461353
Media Volume	186
Agarose Volume	150
Headspace Volume	320
Total # of oxidizable emols/L 1x media	0.123573191
Electron acceptor capacity	0.027344313
Ambient air pressure at location of experiment	0.83
Experimental Temperature	310
Total capsule volume	656
Degree of Reduction for O2	4
Molar concentration of O2 in air (L basis)	0.006836078
Molar concentration of O2 in air (m^3 basis)	6.836078137
Gas Constant	8.314
Partial Pressure of O2	17618.89763
O2 fraction in atmosphere	0.2095
Air pressure	84099.75

README!

Purple (Final Result)	The dilution of 1x media that
Blue (Input Conditions)	Experimental information tha
Green (Part of Final Formula, Don't Change)	Formulas/arrays that directly
Red (Don't Change)	Formulas/values that should t

Units
emols
emols
uL
uL
uL
emols/L media
emol capacity/L air
atm
K
uL
emols/mol O2
mol/L
mol/m^3
J/mol/K
Pascals (N/m^2)
Pascals (N/m^2)

should be used to make the electron donor/acceptor ratio equal to 1
t you need to input, will change based on the conditions of your experiment
contribute to the final volume value
typically be left alone

	Mass per volume for each component (g/L)
Glutamine	0.073
Pyruvate	0.0275
L-Arginine HCl	0.021
Glycine	0.0075
L-Histidine HCl	0.0105
L-Isoleucine	0.02625
L-Leucine	0.02625
L-Lysine HCl	0.365
L-Phenylalanine	0.0165
L-Serine	0.0105
L-Threonine	0.02375
L-Tyrosine	0.0300925
L-Valine	0.0235
Adenine	0
Uracil	0
Cytosine	0
Guanine	0
Glucose	0
Sodium Citrate	0
L-Alanine	0
L-Asparagine	0
L-Aspartic Acid	0
L-Glutamic Acid	0
L-Proline	0
L-Cystine	0
L-Methionine	0
L-Tryptophan	0

MW (g/mol) of each component	# of carbon moles in each component
146	5
88.06	3
211	6
75	2
210	6
131	6
131	6
183	6
165	9
105	3
119	4
181	9
117	5
135	5
112	4
111	4
151	5
180	6
258	6
89	3
132	4
133	4
147	5
115	5
240	
149	
204	11

Degree of reduction for each component
4.8
3.33
5.67
4.5
4.83
5.5
5.5
5.67
4.78
4.33
4.75
4.56
5.4
5
4
4.75
4.6
4
3
5
4.5
3.75
4.2
5
4.73

Molar concentration of organic components
0.0005
0.000312287
9.95261E-05
0.0001
0.00005
0.000200382
0.000200382
0.001994536
0.0001
0.0001
0.00019958
0.000166257
0.000200855
0
0
0
0
0
0
0
0
0
0
0
0
0
0
0
0
0
0
0
0
0
0
0
0
0
0
